# Supplementary material for: Finding the rhythm: Humans exploit nonlinear intrinsic dynamics of compliant systems in periodic interaction tasks
Source: PLoS Comput Biol. 2024 Sep 3;20(9):e1011478. doi: 10.1371/journal.pcbi.1011478 (PMC11398697; doi:10.1371/journal.pcbi.1011478)
Supplement: S1 Table — (PDF) [file pcbi.1011478.s001.pdf]

S1 Supplementary statistical results

to: Finding the rhythm: Humans exploit nonlinear intrinsic dynamics of compliant systems in periodic interaction tasks

Annika Schmidt[1,2,3*], Marion Forano[3,4], Arne Sachtler[1,2], Davide Calzolari[1,2], Bernhard M. Weber[2], David W. Franklin[3,4], Alin Albu-Schäffer[1,2,3]

1 Sensor Based Robotic Systems and Intelligent Assistance Systems, TUM School of Computation, Information and Technology, Technical University of Munich (TUM), Garching, Germany
2 Institute of Robotics and Mechatronics, German Aerospace Center (DLR), Wessling, Germany
3 Munich Institute of Robotics and Machine Intelligence (MIRMI), TUM, Munich, Germany
4 Neuromuscular Diagnostics, TUM School of Medicine and Health, TUM, Munich, Germany

The following tables presents all detailed results for the carried out statistical testing of the obtained experimental data. Since the baseline strategies *BL1-BL3* were deterministic controllers, the here tested samples always refer to the difference of the participant sample and the respective baseline strategy:

$$\Delta BL_i(x) = \boldsymbol{x} - BL_i(x) \ , \tag{1}$$

where $\boldsymbol{x}$ refers to the respective metric vector summarizing the values obtained per participant and $BL_i(x)$ to the corresponding metric value per baseline strategy as presented in Tables 1, 2 and 3 of the manuscript. The obtained differences ΔBL_i were then used for the statistical analysis as described in the method section of the article.

For the data of Experiment 1 a One-way ANOVA was carried out with a post-hoc pairwise comparison using Tukey correction to prove that the tested baseline strategies are different form each other. Following, the obtained metric differences between the different baseline strategies and the participant data were compared against zero with a One-sample t-test as it was assumed that the difference should yield zero if the participant data and one of the baseline strategies align. The t-test analysis was carried out for the data of all conducted Experiments 1-3.

Throughout all presented statistical results, non-significant differences are highlighted by bold p-values.

Table A. One-way ANOVAs on all metrics with the conditions Δ*BL1*, Δ*BL2*, Δ*BL3* of Experiment 1

**metric**	**Source**	**df**	**Sum of Squares**	**Mean Squares**	F	**Prob>F**
**hit score**	Columns	2	208093.33	104046.67	430.04	< 0.001
	Error	57	13791.00	241.95		
	Total	59	221884.33			
**osc. freq.**	Columns	2	9.22	4.609	2094.59	< 0.001
	Error	117	0.26	0.002		
	Total	119	9.48			
**mode metric**	Columns	2	27139.69	13569.84	207.14	< 0.001
	Error	117	7664.88	65.51		
	Total	119	34804.57			
**phase lag**	Columns	2	7.48	3.74	134.99	< 0.001
	Error	57	1.58	0.028		
	Total	59	9.06			
**defl. ratio**	Columns	2	8.24	4.12	1908.62	< 0.001
	Error	117	0.25	0.002		
	Total	119	8.49			

Table B. Multi-comparison between $\Delta BL1$, $\Delta BL2$, $\Delta BL3$ for all metrics of Experiment 1

metric	Group A	Group B	lower limit	A-B	upper limit	p-value p-value
**hit score**	$\Delta BL1$	$\Delta BL2$	-104.837	-93.00	-81.163	< 0.001
	$\Delta BL1$	$\Delta BL3$	37.163	49.00	60.837	< 0.001
	$\Delta BL2$	$\Delta BL3$	130.163	142.00	153.837	< 0.001
**osc. freq.**	$\Delta BL1$	$\Delta BL2$	-0.455	-0.430	-0.405	< 0.001
	$\Delta BL1$	$\Delta BL3$	0.215	0.240	0.265	< 0.001
	$\Delta BL2$	$\Delta BL3$	0.645	0.670	0.695	< 0.001
**mode metric**	$\Delta BL1$	$\Delta BL2$	21.172	25.468	29.765	< 0.001
	$\Delta BL1$	$\Delta BL3$	31.487	35.783	40.080	< 0.001
	$\Delta BL2$	$\Delta BL3$	6.019	10.315	14.612	< 0.001
**phase lag**	$\Delta BL1$	$\Delta BL2$	-0.559	-0.432	-0.306	< 0.001
	$\Delta BL1$	$\Delta BL3$	0.306	0.432	0.559	< 0.001
	$\Delta BL2$	$\Delta BL3$	0.738	0.865	0.992	< 0.001
**defl. ratio**	$\Delta BL1$	$\Delta BL2$	0.575	0.600	0.624	< 0.001
	$\Delta BL1$	$\Delta BL3$	0.473	0.498	0.522	< 0.001
	$\Delta BL2$	$\Delta BL3$	-0.127	-0.102	-0.077	< 0.001

Table C. One-sample t-test statistics for the difference values of all metrics of Experiment 1

			P0		***P90***	
**metric**	**BL**	**df**	**t-stats**	**p-value**	**t-stats**	**p-value**
**hit score**	$\Delta BL1$	19	-9.632	< 0.001	-9.279	< 0.001
	$\Delta BL2$	19	17.107	< 0.001	43.949	< 0.001
	$\Delta BL3$	19	-23.720	< 0.001	-43.325	< 0.001
**osc. freq.**	$\Delta BL1$	19	-1.231	**0.233**	-1.194	**0.246**
	$\Delta BL2$	19	63.592	< 0.001	52.149	< 0.001
	$\Delta BL3$	19	-34.754	< 0.001	-33.115	< 0.001
**mode metric**	$\Delta BL1$	19	2.050	**0.053**	-13.410	< 0.001
	$\Delta BL2$	19	-78.009	< 0.001	-68.309	< 0.001
	$\Delta BL3$	19	-84.442	< 0.001	-107.615	< 0.001
**phase lag**	$\Delta BL1$	19	-0.158	**0.876**	1.298	**0.210**
	$\Delta BL2$	19	11.461	< 0.001	12.160	< 0.001
	$\Delta BL3$	19	-11.776	< 0.001	-9.189	< 0.001
**defl. ratio**	$\Delta BL1$	19	4.787	< 0.001	5.020	< 0.001
	$\Delta BL2$	19	-81.390	< 0.001	-73.854	< 0.001
	$\Delta BL3$	19	-57.168	< 0.001	-69.559	< 0.001

Table D. One-sample t-test applied to the difference values of Experiment 2 for the participant group with decreased target size ($\downarrow r_t$). For the group with increased second link mass ($\uparrow m_2$) only the hit score and oscillation frequency for the newly computed NNM were compared statistically (highlighted in red). Since *BL1-3* were only applied to the original system, results were not expected to match and thus not compared.

			P0		***P90***	
**metric**	**BL**	**df**	**t-stats**	**p-value**	**t-stats**	**p-value**
**hit score**	$NNM_{(\uparrow m_2)}$	9	-10.027	0.00	-5.83	0.00
	Δ*BL1*	9	-8.959	< 0.001	-9.605	< 0.001
	Δ*BL2*	9	6.761	< 0.001	18.305	< 0.001
	Δ*BL3*	9	-17.241	< 0.001	-27.457	< 0.001
**osc. freq.**	$NNM_{(\uparrow m_2)}$	9	1.228	**0.251**	1.69	**0.126**
	Δ*BL1*	9	-4.443	0.002	-3.581	0.006
	Δ*BL2*	9	45.905	< 0.001	22.284	< 0.001
	Δ*BL3*	9	-30.262	< 0.001	-18.989	< 0.001
**mode metric**	Δ*BL1*	9	2.685	**0.025**	-2.707	**0.024**
	Δ*BL2*	9	-62.219	< 0.001	-21.126	< 0.001
	Δ*BL3*	9	-67.426	< 0.001	-34.313	< 0.001
**phase lag**	Δ*BL1*	9	-3.575	0.006	-2.366	**0.042**
	Δ*BL2*	9	7.146	< 0.001	5.696	< 0.001
	Δ*BL3*	9	-14.295	< 0.001	-10.150	< 0.001
**defl. ratio**	Δ*BL1*	9	3.279	**0.010**	2.756	**0.022**
	Δ*BL2*	9	-44.260	< 0.001	-24.225	< 0.001
	Δ*BL3*	9	-30.898	< 0.001	-22.756	< 0.001

Table E. One-sample t-test statistics for the difference values of all metrics of Experiment 3.

			P45	
**metric**	**BL**	**df**	**t-stats**	**p-value**
**hit score**	Δ*BL1*	19	-11.057	< 0.001
	Δ*BL2*	19	24.368	< 0.001
	Δ*BL3*	19	-31.095	< 0.001
**osc. freq.**	Δ*BL1*	19	-2.04	**0.056**
	Δ*BL2*	19	71.802	< 0.001
	Δ*BL3*	19	-43.624	< 0.001
**mode metric**	Δ*BL1*	19	-3.742	0.001
	Δ*BL2*	19	-42.815	< 0.001
	Δ*BL3*	19	-34.333	< 0.001
**phase lag**	Δ*BL1*	19	-1.252	**0.23**
	Δ*BL2*	19	14.422	< 0.001
	Δ*BL3*	19	-13.009	< 0.001
**defl. ratio**	Δ*BL1*	19	3.452	0.002
	Δ*BL2*	19	-102.397	< 0.001
	Δ*BL3*	19	-84.498	< 0.001
