# Supplementary material for: Finding the rhythm: Humans exploit nonlinear intrinsic dynamics of compliant systems in periodic interaction tasks
Source: PLoS Comput Biol. 2024 Sep 3;20(9):e1011478. doi: 10.1371/journal.pcbi.1011478 (PMC11398697; doi:10.1371/journal.pcbi.1011478)
Supplement: Attachment — Submitted filename: ReviewResponse.pdf [file pcbi.1011478.s002.pdf]

Annika Schmidt (an.schmidt@tum.de)
Technical University Munich - CIT - I23
Boltzmannstr. 3
85748 Garching

PLOS Computational Biology
Academic Editor Prof. Dr. Bard Ermentrout
Section Editor Prof. Dr. Daniele Marinazzo

19th January 2024

Response to the Reviewers for the Article Submission
"Finding the rhythm: Humans exploit nonlinear intrinsic dynamics of compliant systems in periodic interaction tasks"

Dear Editors, dear Reviewers,
we would like to express our sincere appreciation for the valuable and constructive comments and suggestions dedicated to our manuscript. Thank you for taking the time and effort to review this work. Your comments have helped us to revise the manuscript significantly, which has greatly improved its quality. This includes fundamental changes in the structure and sectioning, adding more related work and streamlining the text to better present our research. On the following pages, we address the Reviewer comments point-by-point to make our changes as clear as possible. Line references refer to the new clean version of the resubmitted manuscript, since we are aware that the comprehensive changes are hard to follow in the version with the tracked changes. Here, deleted text is red and rewritten parts are marked blue.

Editors - Prof. Dr. Bard Ermentrout and Prof. Dr. Daniele Marinazzo

In light of the reviews, we would like to invite the resubmission of a significantly-revised version that takes into account the reviewers' comments. I apologize for the lengthy review process but it took many attempts to secure two willing reviewers and then there was some delay in the review process due to the complexity of the manuscript. Both reviewers expressed positive views of the paper but also require major revisions. Thank you for your patience.

We thank you for taking the time to find Reviewers with such broad knowledge that they could fully address the many fields our manuscript unites. We have carefully revised our manuscript, taking into account all the useful comments and suggestions of the Reviewers, and are happy to present you now a revised version. For your convenience, we here summarize the main changes:

- We have revised the overall writing style to use less complex and convoluted sentences.
- We have streamlined the Results section to exclude explanations about the applied methods.
- We have extended the literature research to include relevant works suggested by the Reviewers.
- We have improved the methods to reverse-engineer a nonlinear system to explain human behavior.
- We have improved our Figures, especially Figure 1 as suggested by the Reviewers.
- We have added a dedicated Statistics section and clarified the applied comparisons.
- We have heavily reworked the discussion to eliminate identified contradictions and better put our work in perspective with existing literature.
- We have shortened the main part of the manuscript (excluding the Method) by six pages and the complete manuscript by two pages (excluding References).

We believe these changes address all Reviewers' comments to their and your satisfaction such that our revised manuscript can be considered for publication in *PLOS Computational Biology*.

Reviewer #1

The manuscript "Finding the rhythm: Humans exploit nonlinear intrinsic dynamics of compliant systems in periodic interaction tasks" reports experimental evidence that human participants find and exploit efficient and periodic trajectories in the control of a multi-link dynamic system that is prone to chaotic behavior. This is an interesting study with a rich and novel behavioral, applied mathematical, and computational content. The main finding is intuitive but not trivial, and as such would be interesting for a diverse audience. I have three substantial comments, but I believe these can be addressed.
We appreciate that the Reviewer acknowledges our research as rich and novel work and sees it as interesting for a diverse readership. We thankfully received the very constructive comments of the Reviewer and have heavily reworked the manuscript to address the concerns. Please find the individual answers below.

1. *This is much more of a behavioral study than a computational study and as such would be more suitable for the main PLoS than PLoS Comp Bio. It is evident that the study is computationally rich, it's just that the latter part is hidden. Please consider if you could put more of the technical part in the text.*
   Thank you for this remark. We have substantially revised the section "Nonlinear modes of the conservative double pendulum" and added details about the numerical computations of the nonlinear normal modes (NNM). Since the concept of the modes is essential for the carried out user study and the theory is heavily based on algebraic topology and differential geometry, we see *PLOS Computational Biology* as the best fit to ensure the necessary theoretical background of the readership.

2. *I think the manuscript can benefit from re-writing certain parts. It must be possible to convey the necessary information with fewer words. If this is achieved, then it would leave space for specifying the mathematical and computational aspects.*
   We have heavily revised the complete manuscript, especially focusing on using more precise language. The main part of the paper (excluding methods) has been shortened by six pages. Although the complete manuscript has only been shortened by two pages overall (excluding the reference section), we have noticeably extended the section about the mathematical mode background and computations as well as added more relevant literature to better motivate our research [1, 2, 3, 4, 5, 9, 13, 19, 20, 28, 30, 31, 35, 38, 39, 42, 47, 51, 52, 54, 55, 56]. We hope this addresses the Reviewer's concern satisfactorily.

3. *My greatest concern is with the form of the evidence which is not as strong as it could be. The authors state that there is little literature on their topic, "... data-based evidence proving that humans indeed match system dynamics has been lacking." I believe there is a little more than what's listed. Please include literature on feedback resonance and entraining of chaotic dynamics in human motor control. The latter involves a popular problem in the control of chaotic systems, namely stabilizing unstable periodic orbits embedded in a chaotic attractor, and this seems to be similar to the ideas in the present manuscript.*
   We thank the Reviewer for pointing out this shortcoming on our side and pointing us to other relevant literature. We have greatly extended the literature references, including works on human resonance sensitivity [9, 19, 47] and its experimental validation in simple systems [2, 4, 5]. We additionally added sources and background about the human ability to partly learn to predict and stabilize chaos in systems [1, 3, 20, 39, 52, 54, 56]. This background has helped us better position our research in view of the existing literature in the Introduction section [Ln. 1-33] and has helped to detail the motivation for the Baseline strategies used as a comparison for the human

data explained now in the Method section [Ln. 713-726]. Additionally, we set our recent work about nonlinear normal modes in relation to the existing works for periodic patterns in chaotic systems [Ln. 477-489].

4. *It is not obvious how the main hypothesis was tested. In fact, what was the main hypothesis and its null hypothesis? Only after I got to the results did I find that the evidence is in the form of comparing alternative control strategies (BS1, 2, 3). Furthermore, these comparisons do not appear to have been done statistically but just by eyeballing the means ("...is... visually apparent", l. 285-7).*

   Even so, the comparisons are not convincing. For example, it looks like the human is closest to BS1, not to the theoretical mode (Fig 4). This is consistent with Table 1 where it appears that the human participants and BS1 were about equally far from the nonlinear mode reference, implying that participants obeyed control strategy #1. (Is it correct that the mode metric would be 0 if the participant's trajectory was exactly overlapping with the nonlinear mode?)

   We apologize for the unclear statistics and have made it a priority during the revision to be more precise in this regard. We clearly state our hypotheses in the Introduction [Ln. 70-75] and added a separate subsection in the Methods dedicated to the carried out statistics [Line 825]. For clarity, we iterate the null hypotheses in the Results section [Line 112 and 145]. We also clarify the meaning of the mode metric when it is first mentioned in the Results section [Ln. 122-130]. Overall, we have streamlined and reformulated the Results section to explain the mode metric as well as our statistical comparisons more clearly.

   For clarification: The NNM can only be obtained for conservative systems. In an ideal scenario where the frictionless pendulum is deflected from the correct initial positions and the participants would hold the handle perfectly still at the zero position, indeed the pendulum systems should follow their ideal NNM, and the mode metric for participant data would be zero [Ln. 126-129]. However, in this ideal case, the human input would actually be obsolete as the conservative system does not require a control input to stay on the NNM. However, real systems and interactions are not conservative and encounter energy losses, e.g., due to friction. Therefore, the pendulum implementation for the experiment had to encounter minor friction effects [Line 69, 128, 578] such that a controller, e.g., in the form of the human, was needed to continuously drive the system motion. Therefore, the NNM of the conservative system was considered an ideal reference but not expected to be precisely achievable in a damped system interaction [Line 128]. Thus, the mode metric was introduced to quantify the proximity of the excited pendulum motion to the ideal NNM [Ln. 123-129].

   The three baseline strategies *BS1-3* were considered as possible control methods underlying the participants' approach to fulfill the hitting task [Ln. 73-80]. Following known literature about humans' high resonance sensitivity, our null hypothesis was that the participants would intuitively excite intrinsically preferred frequencies and motions like the NNM [Ln. 70-73]. It was expected that the participants would most likely resort to a control strategy similar to *BS1* that excited the eigenfrequency with a sine wave.

   To first validate that the participants indeed excited the resonant pendulum frequency, we subtracted the eigenfrequency determined for the NNM of the conservative case from the oscillation frequency observed for each participant. This sample was then compared against zero with a two-sided t-test ($\alpha = 5\%$). As expected, we did not see a significant difference between the excited participant motions and the predicted eigenfrquency [Line 111 and 829].

   In a second step, we wanted to further confirm the findings by comparing the control approach of the humans with *BS1-3* using the introduced *mode metric* [Line 119]. On the one hand, this metric quantified that the control applied by the participants and *BS1* were closest to the expected ideal NNM of the system compared to *BS2* and *BS3* (Tab. 1). On the other hand, it allowed us to statistically test the hypothesis that the strategy underlying the human motor control was to excite the resonance frequency similar to *BS1*. As with the frequency comparison, we took

the mode metric values of *BS1-3* as offset, which we subtracted from the metric values of each individual participant. Again, we compare the difference against zero with the t-test, as we expected the difference to be zero if a baseline strategy and the human approach align. We found strong significance comparing the human data with *BS2* and *BS3*, indicating that these strategies are fundamentally different than the human strategy. In contrast, we found no significant difference for $\eta_{man}(P0)$ and $\eta_{pos}(P0)$ of *BS1*, indicating that the hypothesis that this strategy is similar to the participants cannot be clearly rejected [Ln. 138-149]. We hope our reasoning and methods become now clear to the Reviewer and are now also easier to follow in the manuscript after reorganizing and rewriting the sections accordingly.

5. *As I see it, the evidence is confirmatory and not very strong. If I understand correctly, the nonlinear mode is also the most energy-efficient trajectory, so it is not surprising that participants converged to it. The main empirical finding reported is that participants could exploit efficient control of nonlinear systems. But this is already stated as a matter of fact at the beginning, so the evidence merely confirms what we already know. The fact that participants found an efficient performance trajectory doesn't tell you much about how they executed it. This is not a bad flaw for a modeling study, it's just important to be explicit about it.*
We thank the Reviewer for pointing out this detail. In the revised version of the manuscript, we have set our research better in the context of existing literature in the Introduction [Ln. 2-33]. We now make clear that we want to collect data-based evidence for expected control principles and behaviors in humans. We have furthermore revised the Discussion to better relate our findings to known motor control principles of the human [Ln. 310-342 and Ln. 378-402]

6. *With relevance to interpreting the significance and results of the study, how hard was it to discover the good trajectories? It appears like this is a learning task, or at least a mix of existing motor skill for control of elastic objects and learning the specific constraints of this object. Were the participants experts in this task from previous studies?*
We purposefully chose the model of a compliant double pendulum as it exhibits dynamics similar to a human limb [Ln. 56-58]. We, therefore, expected the participants to very quickly learn and predict the system dynamics as only an existing motor skill had to be slightly adapted instead of forming a new internal model. This was validated in pilot trials and could also be seen during the training time granted to all participants before the experiment. Within a minute, all participants appeared to be able to predict the pendulum motions and converged to the control strategy observed in the experiment. Comparing the four individual trials per pendulum configuration, the participants did not seem to improve further over time and stayed consistent with their applied control strategy. Thus, learning effects were considered neglectable for the investigated experiment case, which is now addressed in the Discussion [Ln. 317-325]. Nevertheless, the Reviewer points out an important detail, and we are interested in investigating interactions with more complex objects, where the participants first need to learn a new system model in the future.

7. *I think the writing style can be changed so that it's easier to get to the essential information. I didn't understand the task, the nonlinear modes, and the statistical analysis, or lack thereof, until I was on page 9. The Results section is more of a narrative that mixes methods and observations.*
There are also small mistakes that need careful proof-reading for grammar and style.
We thank the Reviewer for this constructive feedback. As a result, we have carefully revised the manuscript. We moved substantial parts of the Results section to the Methods and streamlined the Results to only include relevant information. We have also shortened and rewritten many parts to make the language clearer and more concise. We applied multiple grammar checks and had the manuscript proofread by a native speaker. We expect that this has improved the grammar and style of the manuscript to your satisfaction.

8. *The task, as described early in the paper, is not trivially intuitive for naive readers. Figure 1 helps with illustrating the task, but it could be improved. Could you show the axis of rotation of the joystick?*
As suggested by the Reviewer, Figure 1 was improved accordingly. The illustration of the participant holding the hand was reoriented to align with the red motor link of the virtual pendulum. This should clarify how the handle was moved and how this mapped to the virtual system motion. Additionally, the figure has been extended to show the individual pendulum configurations and visualize the setup and the GUI that the participants saw during the trials. Furthermore, the text description [Ln. 86-92] and the caption have been revised.

9. *How quickly does the pendulum travel? Is it possible to visualize the space of possible trajectories, without cluttering the screen? What would these modes correspond to in an intuitive example with an object used in daily life?*
The speed with which the pendulum moves depends on how the haptic joystick and, thus, the motor link connected to the double pendulum is moved. There is no defined velocity that the pendulum will always converge to, nor is there a specific physical limit implemented. In theory, arbitrary trajectories can be driven by the participants. The system characterization (Fig. 11) should clarify this. The random motion was displayed to show that there is no default path and that the motions can be far away from the intrinsic NNM trajectory. We are not sure how to visualize this in a different way and hope you appreciate our efforts.
Likewise, it is hard to find an intuitive example of a NNM in daily life. The concept of NNM is derived in conservative systems. To achieve the NNM in an interaction with real objects, where friction effects always occur, a controller is needed to compensate for the continuously lost energy and keep the system on the desired energy level and corresponding mode trajectory. A simple example of a mode in a nonlinear system might be that of a child on a swing that uses its legs to inject energy into the system. Considering the connection to the swing as the upper pendulum link and the legs of the child as the lower link, periodic orbits of this nonlinear system are imaginable. The extreme points where the system changes direction represent the turning points of the nonlinear oscillation for a specified energy level. By moving the legs repeatedly at this point, the child adds energy into the swing system to compensate for the energy lost in one period or even to increase the overall energy in the system.
Another graspable example of a nonlinear oscillation might be the different waves that can be excited in a rope. As a system with continuous dynamics that can be represented by infinitely many rigid links, the system is very nonlinear. Fixing one end of the rope and applying periodic oscillations on the other side can excite different waves in the rope depending on the amplitude.
We understand that the topic of nonlinear normal modes might initially not appear intuitive, but we hope our examples make the concept more graspable for the Reviewer.

10. *The labels "init, size, mass" in Table 1 are confusing. Better label as Exp 1, 2, .. or something like that, and explain in the captions that the runs correspond to manipulations of size and mass.*
We thank the Reviewer for their suggestion and have changed the labels in Table 1 accordingly. This should clarify the considered settings and make the comparison easier to understand.

Reviewer #2

This paper is noteworthy as it addresses a new problem: how humans interact with and control a nonlinear underactuated system. The study offers a quantitative demonstration that humans exploit the passive dynamics of nonlinear, underactuated systems. That humans are sensitive to resonance or eigenmodes has been demonstrated in a few studies using simple mass-spring or pendulum systems. However, the eigenmodes of nonlinear systems are much less straightforward, both for the scientist to determine or for the human to sense. Hence, this is an important step forward, both computationally and experimentally. The experiments were thorough and the progression of the three experiments were well justified and alternative hypotheses were tested. As such, this paper is a first demonstration that the notion of 'resonance tuning' in human interactions with physical systems holds for complex underactuated systems that can display chaotic dynamics.
We thank the Reviewer for their encouraging feedback, stressing the relevance of our work. We appreciate the constructive feedback, which has helped us to majorly revise and improve our manuscript. Please find all your concerns addressed individually in the following. Partly, the detailed line comments have been grouped with bigger comments concerning the same topic.

1. *While the experiments are well executed and the results interesting, the manuscript needs a lot more work to be ready for publication. One major drawback is the writing that requires significant revision. Aside from numerous grammatical errors and odd sentence constructions, there is considerable redundancy in content and convoluted sentences sometimes make it hard to understand the meaning. As a broad yardstick, the text can be reduced by at least 30%. I would highly recommend that a native speaker goes over the writing.*
   We have carefully revised the manuscript, reorganized the sections and reformulated large parts of the text to make it more concise. As suggested, we have shortened the main part of the paper excluding methods by 30%, from 20 pages to 14 pages. Although the complete manuscript has only been shortened by two pages (excluding the reference section), we have noticeably extended the section about the mathematical background and computations as requested by the Reviewers. We have also improved the motivation and background with relevant literature.

2. *The researchers cite four of their previous papers [17, 18, 20, 21] [now: 22,23,24,25] that were used to obtain the eigenfrequency and eigenmode of the double pendulum. As the entire study is anchored in this analysis and the analysis is new to the human neuroscience audience, a short explanation of this new approach should be included in an appendix.*
   We have reorganized and streamlined the Results section by moving the details on the mode theory and how the nonlinear normal modes (NNM) are obtained to the Methods [Line 476]. Here, we have substantially extended the background and the underlying theory and methods [specifically Ln. 477-533 and Fig. 8]. We believe that this should better introduce the new concept to the broad readership.

3. *Another major limitation is that the study lacks statistical evaluation of the hypotheses. For example, the results in experiment 1 should statistically compared with the three baseline strategies, such that the conclusion that the resonance strategy best describes human performance is statistically supported. A qualitative description of the mode metric values is not sufficient.*
   Line 367: Provide information what statistical test was conducted that obtained p-values. See comment about absent statistics.
   According to the Reviewer's suggestions, we have added a separate subsection in the Methods [Line 825] that describes the carried out statistics and specifically notes the null hypothesis, which we specifically mention again in the Results section [Line 112 and 145]. Since the baseline strategies *BS1-3* are obtained with a deterministic model controller, we cannot obtain sample data from those

baselines that would allow for direct statistical sample comparison with the participant data. To still compare the participant data to the baseline strategies statistically, the mode metric value of *BS1-3* was subtracted from the participant data. Each obtained offset sample was then tested for the mean value of zero with a two-sided t-test ($\alpha = 5\%$). We expect the sample mean to be zero if a baseline strategy and the human approach align. We found strong significance comparing the human data with *BS2* and *BS3*, indicating that these strategies are clearly different than what the humans did in the experiment. In contrast, we found no significant difference for $\eta_{man}(P0)$ and $\eta_{pos}(P90)$ with *BS1* [Ln. 141-144], indicating that the hypothesis that both strategies are similar cannot be rejected. We believe that the new section dedicated to the performed statistical analysis significantly strengthens the results of our paper.

4. *As a follow-up to this result: the alternative strategies are neither sufficiently motivated nor evaluated. Why did the authors choose these 2 alternative baselines control strategies? Is bang-bang a realistic strategy and something that humans could even reproduce? In addition, does the resonance baseline strategy align with energy minimization? Probably yes, but discuss the closeness to energy minimization. The performance of the alternative baseline strategies is not provided. How many hits are achieved when adopting BS1, BS2 or BS3? Are they similarly efficient in terms of performance (and given the time constraint)? If BS1 is more performance-efficient, it should be mentioned and discussed: humans may first choose performance efficiency before caring about energy.*
We thank the Reviewer for this comment, which we have addressed by improving the motivation for each of the tested baseline strategies and provided supporting literature in the section detailing the baseline strategies [Line 706]. The reasoning for *BS1* is derived from the human resonance sensitivity shown in previous literature [4, 2, 56, 3]. *BS2* was inspired by a reactive control approach that humans apply if they do not have enough feedback to sufficiently predict the system dynamics [13, 35, 33]. The strategy of *BS3* was mainly considered due to results our research group had collected with a previous study [26] and recent findings for hand reaching motions [27]. Although this bang-bang control approach was only derived from the data of one human participant, it has helped to derive a robotic control law that has shown to very efficiently excite intrinsic motions in various robot experiments [58, 59, 60]. It was, therefore, still tested as a possible strategy in the human.
As correctly observed by the Reviewer, we had, however, not reported or discussed the performance of the baseline strategies except *BS1*. We have now addressed this shortcoming. In the Results, we now state the hit score that could ideally be obtained with *BS2* and *BS3* [Ln. 200-204]. Indeed, it shows that with *BS3*, a higher score could have been achieved. We now discuss the reasons why participants nevertheless chose a strategy more similar to *BS1* [Ln. 329-339]. We believe that the main reason has to do with the ability to predict the system dynamics, such that a prospective control approach can be applied. Although not experimentally validated, it is likely that this also translates to a lower mental load and, with this, to a more efficient approach for the human. Thus, it appears that humans primarily care about predictability and the ability to best stabilize a system with considerably low effort instead of trying every possible strategy to maximize performance.
We believe that the extension of our manuscript with those explanations are justified by the better understanding they provide to the reader.

5. *The variable of the phase lag is not sufficiently motivated and explained in the methods.*
We have added background and literature to better motivate our analysis of the phase lag, which is a common measure in system analysis to investigate the input-output response in a forced motion [31]. For linear systems, the value of $\pi/2$ characterizes the phase lag at which resonance typically occurs. At this point, the excitation forces balance the damping forces, which is now also explained in the manuscript [Ln. 171-173]. Thus, we have examined if this was potentially reflected in the participant data, as research shows that the importance of this specific phase lag value holds

only partly true in nonlinear systems [38]. We have discussed the meaning and importance of our observations in the context of general human motor control now in detail in the section "Motor control strategies to excite nonlinear resonance" of the Discussion [Line 343].

6. *Similarly, the mode metric, which is in principle plausible, but the details evade me in the description.* The *mode metric* is based on the principle of *Dynamic Time Warping* (DTW), which is an established tool in the field of gait analysis for comparison of gait cycles of different people. We have added two sources [28, 30] to provide more examples for the interested reader. We have also reformulated the relevant section [Ln. 788-814] to give a more understandable description of the meaning and importance of this metric. We hope this has sufficiently addressed the Reviewer's comment.

7. *Another specific question: Fig 4a (left) shows a significant difference between the theoretical mode and the trajectory for BS2. The only intersecting region lies close to the origin (q1=0, q2=0). Thus, the target locations that can be easily reached using BS1 (because the locations were designed to lie on BS1) would not be reachable with BS2. Can the authors clarify?* We want to emphasize that the target locations were identical for every tested scenario, meaning they were reachable with all baseline strategies *BS1-3*. In Cartesian Space, the plots for the pendulum alternating between the two targets always look similar, and it is hard to see visual differences. For this reason, we initially only plotted the trajectories in joint space in Fig. 2 (Fig. 4 in the original manuscript) to emphasize the differences between the control approaches. The relationship between Cartesian and joint space trajectories becomes more apparent in Fig. 11. When the pendulum moves slowly (Fig. 11, blue: $\omega = 1$), similar to *BS2*, the relative motion of the two joints creates a relatively straight line in joint space (Fig. 11, blue), where the upper and lower pendulum link move together such that the system elasticities are not exploited. With increasing frequency, this line deforms in joint space, although the trajectories in Cartesian Space (Fig. 11, upper row) remain similar. Thus, the targets are reachable with different control approaches and frequencies. To clarify this already in the Results, we added the respective pendulum trajectories excited by *BS1-3* and the participants in Cartesian space (see below) as part of Fig. 2.

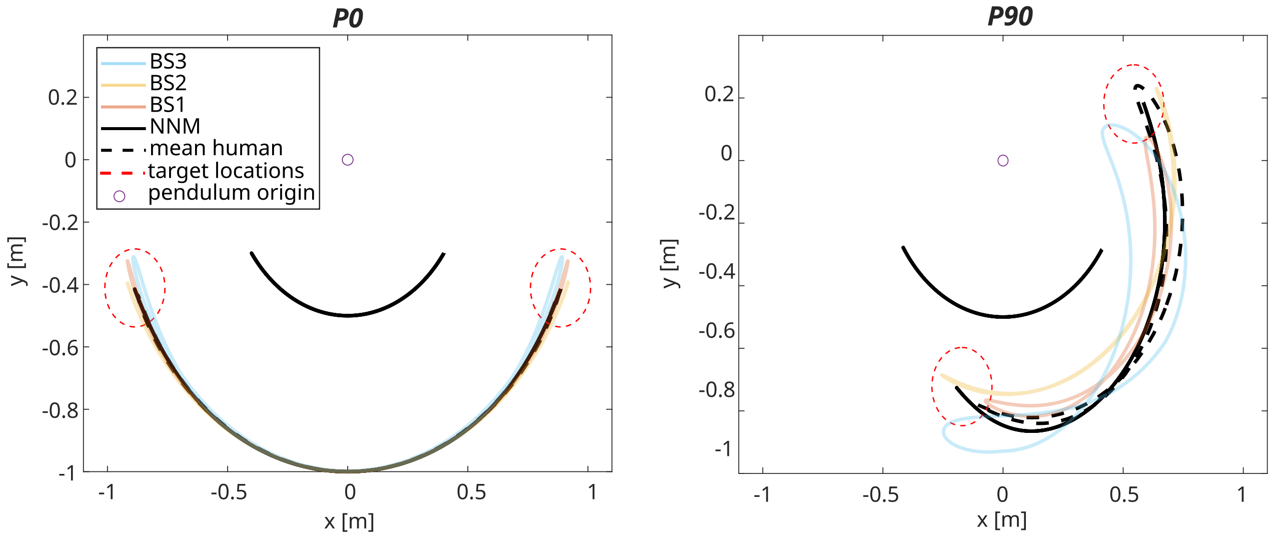

8. *The results of experiment 3 are vital to demonstrate that humans can achieve the task in different ways if the task goals cannot be achieved with the eigenmode. But what are humans do in this case? While reverse-engineering the pendulum that humans effectively implemented, how did they do it? This gets me to a deeper point: the eigenmode analysis is for the conservative system, which is evidently an approximation. Not only does actual pendulum contain damping, the human hand and arm also contribute to the system's properties, adding stiffness and damping. Hence, should we conclude that in experiment 3 the human changes its impedance to overall achieve this different behavior of the double-pendulum? The implications of these results are not sufficiently clear.*
Line 417: when reverse-engineering the double pendulum, it is not clear whether there was more than one solution or whether the mass and spring constant were explicitly chosen for any particular reason. Clarification would be helpful.
We thank the Reviewer for pointing out this shortcoming. We have reworked the applied methods to reverse-engineer a system, such that now only the upper spring stiffness was changed. As suggested by the Reviewer, this is the one system parameter humans can mostly shape. This is now clarified in the Methods section [Ln. 673-678] and also pointed out in the Results [Ln. 260-264]. Additionally, we discuss this observation and the underlying mechanism and influences that might contribute to this control approach in the Discussion now [Ln. 420-463]. This should better reflect and explain the experiment observations and set it in the appropriate context of known human motor control.

9. *Another potentially interesting aspect that may strengthen the data is to examine whether participants are converging on a solution with practice.*
For the investigated experiment, we purposefully chose to use the compliant double pendulum as a system with dynamics that are familiar to the human [Ln. 56-58]. Thus, we expected the participants to learn to predict the system behavior quickly, as the general system dynamics were known and existing internal models only had to be slightly adapted. This was validated in pilot trials and could also be seen during the training time granted to all participants before the experiment. Within a minute, all participants appeared able to predict the pendulum motions and converged to the control strategy observed in the experiment. Comparing the four individual trials per pendulum configuration, the participant did not seem to improve further over time and stayed consistent with their applied control strategy. Thus, learning effects were considered neglectable for the investigated experiment case, which is now addressed in the Discussion [Ln. 317-325]. However, we agree with the Reviewer that it would be interesting to investigate human strategies and their ability to adapt to new systems in more detail. Thus, we aim to address this in a further study with a wider variety of tested system parameters and possibly include more complex systems with more unfamiliar behavior. We hope the Reviewer understands our reasoning for not including a dedicated analysis of learning in the present manuscript.

10. *The discussion needs to elevate the results to a higher level why and how humans interact with nonlinear dynamic systems.*
We have carefully revised the discussion and shifted the focus to set our findings into perspective with existing work. Since the human dynamics as well as most objects in everyday interactions are usually nonlinear, the evidence collected in our research can support further research to eventually better understand human motor control. In the Discussion, we now expand on the underlying reasons why humans might intuitively choose to excite resonance also in nonlinear systems, as this has been shown to make system behavior easier predictable [9]. We dedicate a section of the discussion [Line 288] to why humans might prefer predictability in periodic interactions and how prospective control might be beneficial, especially in a task that is inclined towards speed instead of accuracy. We also expanded on the meaning of the phase lag that the individual participants applied and motivated why different participants might be successful with different values [Line 343]. Eventually, we expand on the changed performance for the experiment task variation with decreased

target sizes and target relocation away from the NNM [Line 413]. We provide theories of why and how humans adapt in these cases, which is also further detailed in the items 8 and 18 of this review answer.

11. *Line 22-23: No direct connection between the text and the study in ref [8].*
Line 22-23: Says "humans might excite the simple intrinsic modes of the system to hit a target". The concept of modes has not been introduced, making this statement irrelevant and confusing.
Line 36-37 : The relevance of [12, 13] to the prior text is unclear. Must briefly discuss the cited work to give better content.
We have addressed all the above points with a major revision of the Introduction, such that these confusing sentences no longer appear in the revised manuscript. Instead, we now set our work better in the context of existing literature to explain our motivation for the research. We hope this has improved the Introduction and aided the clarity and understanding.

12. *Line 127: What creates the different energy levels? They introduce this later, but I believe this caused lots of confusion when interpreting figure 2 and the definitions of eigenmanifold, eigenmode, etc.*
Line 170: Why was 2.5J energy level chosen? Why not a different energy level? Was it the total physical energy of the conservative system? A clearer description of what the energy level physically means would be informative.
We acknowledge that the mentioning of the energy level might be confusing and complicated following of the obtained results. The reason why the energy level is important in our experiment is that the orbits of the NNM change their shape with energy. However, the exact value for the energy level used in the experiment comparison was arbitrarily chosen. Although the mode trajectory would have looked slightly different for a higher or lower energy level, likewise, the target locations would have been different, such that the participant data would probably also have adjusted accordingly. To avoid confusion, we decided not to detail the considered energy level in the Introduction and Results. Instead, we expanded the theory on nonlinear normal modes in the Methods section [Line 476] and emphasized here the meaning and relevance of the energy levels. Specifically, we adjusted Figure 8 (Fig. 2 in the original manuscript) to improve the understanding of how NNMs change with increasing energy levels. Only in this section do we now state the specific level used in the experiment [Line 560]. This should improve the readability of our manuscript without sacrificing clarity.

13. *Ln 195-196: What is different from the initial pilot experiments that led participants to not use a position control strategy? Was the same observation made in the reported experiments during the early trials?*
The reason that the participants applied a control approach in the training phase that resembled position control was most likely that they needed this initial time to test out the system and learn the dynamics. Most likely, they initially applied a more reactive control strategy more dependent on sensory feedback before they could sufficiently predict the system behavior and apply the strategy that was seen in the experiment. This is now also mentioned when introducing *BS2* [Ln. 732-740] and specifically addressed in the Discussion [Ln. 310-320].

14. *Line 202: Insufficient justification for use of bang-bang control in this study. Are there existing studies that suggest humans use such control strategy?*
The main reason for investigating the bang-bang control approach (*BS3*) as an alternative baseline strategy was a previous study on a similar setup [26] and the findings that this approach can predict hand reaching motions [27]. Additionally, this control approach has been shown to be very efficient [58] to effectively excite intrinsic motions in robotic experiments [59, 60]. We therefore considered this bang-bang approach to be a possible method a human could choose to excite intrinsic resonant

motions in this way, and we, therefore, selected it as one of the baseline strategies. We provide the same reasoning in the Introduction [Line 79] and the Method section [Ln. 745-750].

15. *Line 266-277: Extremely dense paragraph. Hard to follow.*
We have carefully rephrased and shortened the paragraph by excluding unnecessary details [Ln. 130-133]. This should aid the readability and understanding by focusing on the most important aspect.

16. *Ln 444-448: The reported behavior seems to indicate an eigenmode: small input leading to a large output and the oscillatory behavior traverses the same trajectory repeatedly in the q1-q2 space. Can the authors clarify if they would call this an eigenmode?*
The described behavior of the participants only moving the input handle little with comparatively large output amplitudes indicates that resonance is excited. As the NNM of nonlinear systems is similar to the eigenmode known in linear systems [Ln. 38-40], this control approach was expected. However, we made this connection and the relation to resonance clearer in the new version of the manuscript. We extended the background on human resonance sensitivity [9, 19, 47] and its experimental validation in simple systems [2, 4, 5] to better outline the context of the investigated research and nonlinear modes in the Introduction. We also discuss the applied handle motions and the overall strategy in more detail now [Line 343]. We hope this clarifies the Reviewer's question and better addresses it in the manuscript.

17. *Line 505: This study is not the first one to show how humans can intuitively interact with nonlinear dynamic systems (cf Turvey's studies, that should be cited as well). But this study digs deeper into this interaction by investigating interaction with more complex systems (double pendulum).*
We thank the Reviewer for pointing us in the direction of more relevant literature in this field. We have accordingly extended the reference list [1, 2, 3, 4, 5, 9, 13, 19, 20, 28, 30, 31, 35, 38, 39, 42, 47, 51, 52, 54, 55, 56] and revised the Introduction and Discussion accordingly. This now includes research about human resonance sensitivity and the experimental validation of such. Additionally, we incorporated more literature on human motor control to put our experiment findings better in the context of existing knowledge.

18. *Ln 517-524: It is unclear how the discussion point (1) is relevant to the argument made about "why humans choose to exploit the inherent dynamics" of the mechanical system. Are the authors trying to convey that participants excite the system at the eigenfrequency (indicating exploiting dynamics) since the accuracy requirement was not high enough. It counters the statement from experiment 2 that participants still exploited the intrinsic dynamics of the system when the accuracy requirements were increased. Maybe the authors can rephrase and clarify this paragraph.*
Ln 573-577: It is first claimed that the strategy was not altered, and the following sentence starts by "accordingly, the participants might likely alter their strategy [...]" it seems contradictory. Please clarify.
We agree with the reviewer about the contradiction in the referenced statements. We have carefully revised the Discussion section and no longer make this statement. We now specifically address the significant difference seen for the excited frequency in Experiment 2. Similar to the observations in Experiment 3 (see Comment No. 8), we hypothesize that the change in behavior could be due to people intuitively adjusting their arm or wrist stiffness to better meet the demands of the task [Ln. 420-463].

19. *Line 585: what does this head mean? (now [Line 343])*
The Discussion has been carefully revised and this section name does no longer appear. Instead, the section has been renamed "Different motor control strategies excite nonlinear resonance".

20. *Ln 595-597: this passage starts by "the experiment data suggests that a phase lag around 0.6pi might be the best choice", but the next sentence continues with "the present data is inconclusive".*

Please clarify. It is unclear how the phase lag can be independently chosen by the participants. Should the phase lag not naturally arise with the type of excitation i.e., depend on the nonlinear phase response. For instance, with a linear system, each excitation frequency is also associated with a phase lag. Can the authors clarify if it is possible to dissociate the relationship between excitation frequency and phase lag for a nonlinear system?
Firstly, we would like to refer the Reviewer to Item 5, where we have now added background and literature to better motivate our interest and expectations for the phase lag. We have clarified why a phase lag around $\pi/2$ was expected as in linear systems this value usually characterized resonance. However, we now also point out that the participants were able to excite resonance with different phase lag values. This aligns with recent findings from literature [38] indicating that not one single phase lag value can be associated to resonance in nonlinear systems. This is also discussed separately in the manuscript now [Line 343]. Nonetheless, no clearly significant correlation of the applied phase lag over all performance metrics such as hit score and mode metric could be found. To investigate this in more detail, a bigger variety of tested system parameters is probably needed, especially considering systems with higher friction such that the moment in which energy is injected might become more important. We plan to address this in future studies, which is also mentioned in the manuscript [Ln. 409-412].

21. *Figure 1: The perspective on the hand in relation to the monitor display of the double pendulum makes it hard to get a sense of the task.*
We have changed the perspective of the human hand holding the handle to align with the sketch of the pendulum definitions in the experiment setup to clarify the arrangement. Additionally, we have extended the Figure to include a sketch of the layout shown to the participant during the experiment. We hope this improves the understanding of the experiment task and setup.

22. *Figure 2 (now Figure 8), panel (d): What is plotted on the y-axis? I understand that this compares with panel (e). Maybe adding better labels for the axes and colored lines within the figure and not buried in the wall of text that is the caption.*
We have extended the explanation and derivation of the nonlinear normal modes in the Method sections. For this, we also replaced the previous panels (d) and (e), which were only sketches to qualitatively explain the concept of NNM with the specific mode trajectories for different energy levels of the *P90* pendulum configuration. We also included a legend to improve readability. We hope that this adjustment, together with the extended theory section, improves the understanding of NNM and how they develop for different energy levels.

23. *Figure 3 (now Figure 9): the gray straight lines in panels (a) and (b) are not explained. I think looking at panels (c) and (d) first before (a) and (b) would be easier to interpret. Switching the order of the panels would be easier to interpret.*
We have implemented the Reviewer's suggestions for this figure. We have removed the grey line in panel (a) and (b), which represents the second mode of the system. This mode is exemplarily introduced and explained to show the concept of the *Generator* and NNM in Figure 8, but was neglected in Figure 9, as it is not considered in the carried out experiment. We hope that in this way confusion can be avoided. Additionally, we swapped the panels to first show the Cartesian space plot for each pendulum configuration and only then add the joint space next to it.

24. *Figure 6 (now Figure 4): the pendulum symbol P0 and P90 is different - legacy?*
We thank the Reviewer for pointing out this inconsistency. We have changed the symbols to *P0* and *P90* in the revised manuscript version.

25. *Figure 9 (now Figure 12), panel (a): the gray line as in Figure 3 are not explained. Also, the order of the panel labeling is a little confusing.*

As in Figure 9 (Figure 3 in the original manuscript), we have excluded the grey line showing the second system mode in panel (a). The alignment of the plots has also been changed to clarify that (a) includes both the Cartesian and the joint space plots. We hope this improves the understanding of this figure.

26. *Figure 10 (now Figure 7): the label says "adjusted mode" - please explain.*
We have adjusted the mode label to "NNM $k_1 = 6$" to make it immediately obvious that only the stiffness value of the upper spring k_1 was changed to $6\,\mathrm{N\,m\,rad^{-1}}$. This should clarify how the adjusted NNM was obtained and how the orbit trajectory changed in comparison to the original system parameters.

We believe that the current version of the paper addresses all questions of the reviewers and look forward to hearing back from the committee.

References

[1] Takagi A, Furuta R, Saetia S, Yoshimura N, Koike Y, Minati L. Behavioral and physiological correlates of kinetically tracking a chaotic target. Plos one. 2020;15(9):e0239471.

[2] Dotov DG, Stephen DG, Frank TD. Variability of Uni-Manual Pendulum Oscillation at and away from Resonance. In: Studies in Perception and Action X: Fifteenth International Conference on Perception and Action. Psychology Press; 2009. p. 8.

[3] Pitti A, Lungarella M, Kuniyoshi Y. Chapter 15 Quantification of Emergent Behaviors Induced by Feedback Resonance of Chaos. In: Recent Advances in Artificial Life. World Scientific; 2005. p. 199–213.

[4] Turvey M, Rosenblum LD, Schmidt R, Kugler PN. Fluctuations and phase symmetry in coordinated rhythmic movements. Journal of Experimental Psychology: Human Perception and Performance. 1986;12(4):564.

[5] Riley MA, Turvey MT. Variability and determinism in motor behavior. Journal of motor behavior. 2002;34(2):99–125.

[9] Goodman L, Riley MA, Mitra S, Turvey M. Advantages of rhythmic movements at resonance: minimal active degrees of freedom, minimal noise, and maximal predictability. Journal of motor behavior. 2000;32(1):3–8.

[13] Kuznetsov NA, Riley MA. Dynamics of Motor Performance in Visually Guided Force Production. Studies in Perception and Action X. 2009; p. 26.

[19] Pitti A, Niiyama R, Kuniyoshi Y. Creating and modulating rhythms by controlling the physics of the body. Autonomous Robots. 2010;28:317–329.

[20] Kuznetsov AP, Migunova NA, Sataev IR, Sedova YV, Turukina LV. From chaos to quasi-periodicity. Regular and Chaotic Dynamics. 2015;20:189–204.

[26] Lakatos D, Petit F, Albu-Schäffer A. Nonlinear oscillations for cyclic movements in human and robotic arms. IEEE Transactions on Robotics. 2014;30(4):865–879.

[27] Leib R, Russo M, d'Avella A, Nisky I. A bang-bang control model predicts the triphasic muscles activity during hand reaching. Journal of Neurophysiology. 2020;124(1):295–304

[28] Boulgouris NV, Plataniotis KN, Hatzinakos D. Gait recognition using dynamic time warping. In: IEEE 6th Workshop on Multimedia Signal Processing, 2004. IEEE;2004. p. 263–266.

[30] Switonski A, Josinski H, Wojciechowski K. Dynamic time warping in classification and selection of motion capture data. Multidimensional Systems and Signal Processing. 2019;30:1437–1468.

[31] Géradin M, Rixen DJ. Mechanical vibrations: theory and application to structural dynamics. John Wiley & Sons; 2014.

[33] Franklin S, Franklin DW. Feedback gains modulate with internal model uncertainty. Neurons, Behavior, Data analysis, and Theory. 2021.

[35] Wolpert DM, Flanagan JR. Motor prediction. Current biology. 2001;11(18):R729–R732.

[38] Volvert M, Kerschen G. Phase resonance nonlinear modes of mechanical systems. Journal of Sound and Vibration. 2021;511:116355.

[39] Dotov D, Froese T. Entraining chaotic dynamics: a novel movement sonification paradigm could promote generalization. Human movement science. 2018;61:27–41.

[42] Seidler RD, Noll DC, Thiers G. Feedforward and feedback processes in motor control. Neuroimage. 2004;22(4):1775–1783.

[47] Dotov DG, Cochen de Cock V, Geny C, Ihalainen P, Moens B, Leman M, et al. The role of interaction and predictability in the spontaneous entrainment of movement. Journal of Experimental Psychology: General. 2019;148(6):1041.

[51] Strogatz S. Nonlinear dynamics and chaos: with applications to physics, biology, chemistry, and engineering, 2nd edn Boulder. CO: Westview Press[Google Scholar]. 2015;.

[52] Pecora LM, Carroll TL. Synchronization in chaotic systems. Physical review letters. 1990;64(8):821.

[54] Harvey K. General Principles of Neurorobotic Models Employing Entrainment and Chaos Control. Frontiers in Neurorobotics. 2019;13:32.

[55] Vaschillo EG, Vaschillo B, Lehrer PM. Characteristics of resonance in heart rate variability stimulated by biofeedback. Applied psychophysiology and biofeedback. 2006;31:129–142.

[56] Pitti A, Lungarella M, Kuniyoshi Y. Exploration of natural dynamics through resonance and chaos. In: International Conference on Intelligent Autonomous Systems (IAS-9); 2006. p. 558–565.

[58] Stratmann P, Lakatos D, Özparpucu MC, Albu-Schäffer A. Legged Elastic Multibody Systems: Adjusting Limit Cycles to Close-to-Optimal Energy Efficiency. IEEE Robotics and Automation Letters. 2017;2(2):436–443.

[59] Della Santina C, Lakatos D, Bicchi A, Albu-Schäffer A. Using Nonlinear Normal Modes for Execution of Efficient Cyclic Motions in Articulated Soft Robots. In: Siciliano B, Laschi C, Khatib O, editors. Experimental Robotics. vol. 19. Springer International Publishing; 2021. p. 566–575.

[60] Schmidt A, Feldotto B, Gumpert T, Seidel D, Albu-Schäffer A, Stratmann P. Adapting Highly-Dynamic Compliant Movements to Changing Environments: A Benchmark Comparison of Reflex-vs. CPG-Based Control Strategies. Frontiers in Neurorobotics. 2021;15.
