# Supplementary material for: Finding the rhythm: Humans exploit nonlinear intrinsic dynamics of compliant systems in periodic interaction tasks
Source: PLoS Comput Biol. 2024 Sep 3;20(9):e1011478. doi: 10.1371/journal.pcbi.1011478 (PMC11398697; doi:10.1371/journal.pcbi.1011478)
Supplement: Attachment — Submitted filename: Schmidt.et.al.PLoS.CompBio.pdf [file pcbi.1011478.s003.pdf]

Review of Schmidt, Forano, Sachtler, Calzolari, Franklin, Albu-Schäffer: Finding the rhythm: Humans exploit nonlinear intrinsic dynamics of compliant systems in periodic interaction tasks

The authors provided an extensive revision of their manuscript in response to our comments. The re-writing of the manuscript has significantly improved readability. The authors have further strengthened some of their arguments by adding references and clarifying metrics, figures, and analyses. While this revised manuscript is a significant step forward, there is still a long list of comments and suggestions to further improve the manuscript. While the list is long, the study is scientifically valuable and, with more work, will hopefully become more rigorous in its presentation.

Major Comments

1) While the writing has improved, many passages, especially in the discussion, are still quite wordy and 'unconstrained'. Tightening the language would improve readability. Some choices of words and phrases still indicate that a native speaker should go over the text.

2) The results are inconsistently reported with seemingly select statistics and the results of Experiment 2 are only reported visually. The human data obtained in the three experiments should be consistently compared with the three hypothesized strategies. The authors have introduced three metrics: task success, mode metric and phase lag. For each experiment, each of these metrics can be compared with the three hypothesized strategies. Coupled with the result that for example the mode metric was lowest for BS1, it can be inferred that participant trajectories were closest to the ideal NNM.

3) The experimental task is still unclear and difficult to understand, especially as different aspects of it are shown in three different figures. While the task variations are clear (reduced target size, etc.), the way the task is carried out by a human remains hard to visualize. For example, did the subject move their arm laterally to move the apparatus, or did they rotate the wrist, and in what direction? Provide more description on exactly how the user manipulated the apparatus. Combining at least the first and second figure will help.

4) The discussion of the control strategies is sometimes contradictory. The frequencies by themselves are not sufficient indicators of whether participants perform feedback (reactive) or feedforward control (prospective) as the authors also argue that participants could be modulating their arm stiffness to shift the NNM mode to lower frequencies. The more relevant difference between BS1 and BS2 is that BS1 requires low forces (low input, thus resonance and using NNM) and BS2 requires greater forces (large input, not exploiting resonance and not using NNM). It may be beneficial to have a different name for BS2. In Experiment 2, the authors argue that participants shifted the oscillation frequency farther away from BS1 to become more accurate. Does this not negate the hypothesis of the authors? If participants want to be more accurate, they should more accurately match their oscillation frequency to the "resonant" frequency of the system.

5) The discussion section talks about aspects of the data that were not reported in the results. The sinusoidal shape on the human trajectory, in line with the BS1 strategy and the initial explorative movements that were not shown in the results. These data should either be shown or eliminate reference to these data aspects in the discussion.

6) The target hit score seems somewhat biased. It is argued that overswing would alter the energy level and therefore would confound the comparison with the NNM. Do undershoots or missed targets not lead to a lower energy mode? If so, should they not also count as -1 as the overswings? A score report that splits between hits and misses, both over and undershoot may help clarify this. A different count of the hits may also reveal different patterns in the correlations with the phase lag.

7) The two mode metrics seem to obtain different results when compared with the NNMs. This is confusing. It appears that the mode metric of the full state space is the more complete one, hence the one that should be reported. While some differences may be less favorable for the hypothesized

strategy, I assume the new suggested statistical comparison will render new and likely supportive results for BS1.

8) Overall, the concept of energy levels must be discussed in more detail. It is still unclear to me how different energy levels are achieved/excited. Is it by adjusting the initial angles of the pendulum, or by introducing a non-zero velocity as initial condition or?

9) The simulations for BS2 and BS3 should be explained more prominently as they serve as reference or alternative hypothesis throughout the paper.

Detailed Comments (following the text):

Introduction

Line 5 onwards: I appreciate the authors to better situate their work in existent understanding of human motor control. As I am keenly interested in this area of study, I probed into the cited papers. Unfortunately, I found some of the new references non-optimal. For example, amongst the references for predicting and following chaotic behavior, reference 2 is a non-refereed 4-page extended abstract. A similar extended abstract is cited later. For a journal like PLoS Comp Biology, it may be better to use peer-reviewed papers, especially in the introduction. The paper by Goodman et al (ref 9) seems more to the point here. Ref 5 is not really on resonance, may be better Kugler and Turvey (1987) and Raftery, Cusumano, & Sternad (2008). I also do not think that reference 7 is a good reference for resonance and dynamic stability.

Line 16: This point was also made by Maurice, Hogan, Sternad (2018).

Line 20: Reference 20 is again an unrefereed extended abstract, in the current context, 'prospective control' appears misleading as it was only an experimental manipulation, not a reliable assessment or simulation of prospective control. See also Sharif Razavian, Sadeghi, et al (2023) for this point.

Line 26: See also Nasseroleslami, Hasson, Sternad (2014) for the comparison between predictability, stability and smoothness.

Line 67, Figure 1: I fail to see the blue, orange and yellow configuration in the figure? Only the targets are colored and the pendulum links are white. Why not simply showing three pendulums separately? Also make the text follow the figure: first explain the left panel. Also, is the hand orientation really matched with the hand figure? Then, the hand would overlap or 'collide' with the double pendulum. Would it not make more sense to have the hand coming from the right to hold the red handle? Unnecessary: the hand looks as if it is manipulated by the left hand. Are the indicator panels for the remaining time and target hit score really as large? Figure 12 may be combined with Figure 1 to not distribute different illustrations of the experiment throughout the paper.

Line 75: I would not call the strategies 'baseline strategies', later abbreviated to BS. First, the acronym BS has negative connotations in English and American slang. I suggest to call them hypothesized strategy for the resonance tuning one, and alternative strategies for the other two. Or just short names. I also have reservations about strategy 2 as explained in the general comment above.

Line 81: The two metrics are not a contrast, delete 'on one hand' and 'other hand'.

Results

Line 105: It may help readability to summarize the experimental task for the subjects, including the duration and scoring achieved. Also, state the exact conditions on Exp 1, 2, 3. It would also help to have a brief summary of the simulated strategies, BS1, 2, 3.

Line 112: It is typically useful to first state how participants achieved the task. In this case, the explicit instruction was to hit targets. Hence reporting target scores would be good. May be also list overswings and hits separately so that the reader gets an overall idea how difficult or easy the task was.

I also would not refer to the resonance strategy as null hypothesis, because null hypothesis denotes the claim that the effect being studied does not exist or when there is no relationship between two conditions.

L 116: Which statistical comparison was applied? A linear mixed model with the two oscillations or did you use t-tests for each of the two comparisons? If yes, them report t statistics with degrees of freedom and Bonferroni adjustments. See comment on statistics. Table 1: use the same labels for the pendulums.

Line 131: Which data were entered to calculate the means and standard deviations? All trials and all s subjects? Again, report the statistical results.

Why were two mode metrics calculated, one for position and one for the full state space? The results are very similar and only one of the two is needed. I suggest the more complete metric for the full state space. Figure 2 does not have red traces?

Line 139-146: The statistical evaluations for these important tests are obscure and the conclusion that there is no significant difference, although P90 does differ, is sketchy. There are two significant p-values, which suggest that participants' dynamics are somehow different from BS1 (in position for P0 and over the full manifold for P90). While participants are closer to BS1 than to the other strategies, they still differ when using this analysis. Suggestion: compare the subjects' data (include 3 trials as separate estimates) using a linear mixed model with the 3 hypothesized strategies. The mode metric is likely to be lowest for BS1 as hypothesized.

Table 1: Comparing values of BS1 and Exp1, it seems that participants were able to perform the task "better" than BS1 based on the metrics introduced. Could this suggest that even BS1 is not the correct baseline strategy to compare against participant strategy? I think it is important for the authors to touch on this in the discussion.

Figure 2: target locations are ellipses indicating different axes. Better make axes the same +1 to -1m to give a more realistic impression. May be the same for joint space depiction.

The section on applied handle motion is hard to follow as its rationale was not introduced or prepared in any way. See more detailed comments in the following:

Line 155-157: Sentence 'Therefore …require a control input' is unclear. I understand that if the handle is displaced, the conservative system would continue with the handle moving accordingly. Would this not imply that the larger handle deflection and sinusoidal motion with the initial amplitude needs to continue? Alternatively, I understand that at resonance the input amplitude can be small. In this case, the friction in the pendulum requires continued input. These three observations do not quite converge.

Line 160: If 1.55 rad is the amplitude, then it would be better to say that the amplitude of P0 was 1.5 rad, not moved more … I do not see the numbers how this amounts to 14% or 17% larger amplitude. Is there an explanation for the offset of the human strategy compared to the simulated strategy? Also, these values are 0.5pi, as stated below. Relate this number to .5pi before when the numbers are presented.

Line 179: past tense is confusing here, the phase will be compared to task success (task success should be mentioned first in the results).

Lines 179-192: correlations were illustrated in Figure 6 with participants ranked by either the two score metrics with a linear trend line, paired with values of the phase lag highlighted by a separate linear trend line. This is a confusing way to report correlation. Illustrate correlation analysis via scatter plots of X vs. Y. Consider also adding confidence bands.

Line 188: both correlation coefficient and p-value are relatively far from significance, rephrase.

Line 192: the highest value was reported to be .74pi?

Line 207: replace ‘stretched’ pendulum with ‘extended’ pendulum here and below. Do the reported hit numbers for the participants include both hits and overswing? Please report task success with all three occurrences.

Lines 217-220: If I understand this correctly, in order to become more accurate, participants shifted the oscillation frequency farther away from BS1? Does this not negate the hypothesis of the authors? If participants want to be more accurate, they should more accurately match their oscillation frequency to the “resonant” frequency of the system.

Also, the tests should again be corrected and include 3 trials.

Figures 5, 6 and 7: Axis labels saying position or velocity are redundant. Could be removed to reduce clutter. Also, for the two left panels, y axis could be θ [rad]. What denotes the green color in Figure 5? Caption word over all correct to ‘overall’. Report hit rate consistently as number, not percent.

Line 245: “..a setup is unlikely, ‘such that’ we used an additional..”. ‘such that’ is not English here

Line 275: “..data supporting the ‘intuitive’ hypothesis..” The hypotheses tested here are not purely intuitive but theoretically and quantitatively motivated.

The results of Experiment 2 also warrant a quantitative comparison with the mode metric. As it stands, the results are only reported visually.

Lines 250-270: the results of this experimental condition should again be compared to the three hypothesized strategies, using the three descriptive metrics: target hits, mode metric, and phase lag.

Discussion

Line 304: the fact that human handle trajectories were close to sinusoidal, similar to the sinusoidal input of BS1 was not really dealt with and tested in the results. If this is a strong observation, this should be tested.

Lines 310-322: In the rest of the manuscript, the “training” period was completely omitted in the analysis. Yet, data from that period are discussed here as though the reader has been previously presented some of the trends and behaviors discussed. For example, on line 318 it is mentioned “the learning curve during the training period appeared very steep”. If this is an important point that needs to be discussed, then perhaps a figure should be added to show the mentioned learning curve. The way the text stands now, it feels very vague and unsubstantiated.

Line 327: The statement that subjects adopted a prospective control approach appears bold as it is only based on the assumption that resonance behavior is more predictable as reported in another study. Such relations need to be reported more carefully as conjectures, rather than facts.

Lines 310-342: this is quite lengthy and could be made more succinct.

Line 348: Could I be that the close location of the handle on the screen biased the subject as otherwise there may be an overlap?

Line 359: rephrase ‘dedicated’

Line 365: this discussion is confusing: it is proposed that the visual display biased subjects and that this then causes deviations from the sinusoidal trajectory shape. This contradicts what was said above.

Line 398-411: these statements about chaotic motion and control are confusing. First, the human data were not analyzed whether they revealed chaotic features. They were only compared to the NNM. The discussion about chaotic control or predicting chaotic evolution appear disconnected from the results.

Line 413-432: I am not convinced by the argument made in this section explaining why participants slightly reduced their movement frequency when the target size was reduced. Fitt’s Law does not seem like a valid argument to be made here. While it is true that humans often obey the speed-accuracy trade-

off, this is not always the case as conflicting constraints may suppress it: Kelso, Southard, Goodman, 1979). But if speed-accuracy trade-off is the primary driving factor, then BS2 must be the best strategy as it is the slowest, hence the most accurate strategy.

Line 457: sentence grammar 'it is not only ..

Line 460: Here resonance sensitivity and prospective control is equated. This is not correct.

Methods

Figure 8a: increase the turning points (hollow dots) that are hardly visible to ease understanding of the figure.

Figure caption: delete 'a' in line 2, change second d) to e)

Line 541: exemplary, … not English, change to: As an example,..

Also, it is nearly impossible to see these dotted lines on the figure, if they are important to be pointed out, then perhaps use a different color to plot them.

Figure 9, caption: joint space is depicted on the right, not left

Line 561: "level E =2.5 J that is arbitrarily chosen"
In the previous version of the manuscript, the justification for this choice of energy level was based on "human preference". Knowing that, to say a value was arbitrarily chosen seems like the authors did not want to spend an extra sentence giving a deeper and more satisfying justification for this choice.

Lines 561-562: "In this investigation, only the first, more stable mode will be considered"
How was 'more stable' quantified and identified?

Line 581: "two links with ball geometry were added as targets that had to be reached." I do not understand whether the ball geometry refers to the links or the targets, rephrase

Line 583: This is not a collision with force, change collision to the link reached or hit the target

Lines 598-600: "In the case of the random control, the amplitude A and frequency ω were pseudo-randomly resampled every 0.1 s) with $A \sim U(1, 10)$ and $\omega \sim U(0, 10)$ rad s^{-1}."
For $A \sim U(1, 10)$, does this mean that the amplitude of oscillation could be 10 radians? Which means multiple complete rotations (2pi) of the link?

Figure 12: While these figures now make understanding the task easier, I wonder what the hand motion was: wrist flexion and extension in the general position shown in Figure 12c? Or was it a forward/backward movement? How did this match with the visual display of the red handle?

The experiment figure would be better combined as the initial figure in the introduction.

Line 675: "this specific parameter" there is no parameter value mentioned yet. I suppose you mean the value mentioned in the next sentence? Also, the sentence does not give a real rationale: if humans add their own stiffness, why do you need a stiffness in the virtual motor link?

Line 704-705: "After one completed experiment, the participants filled out a NASA TLX-inspired questionnaire." Why include this if the questionnaire is not discussed anywhere else in the manuscript?

Line 706: I would rephrase baseline strategies to hypothesized strategy and alternative strategies, HS and AS1 and AS2. Or give is short names. To label strategies BS is too close to slang for 'bull shit' (sorry).

Line 725: a reference for 'humans seek predictability' is Nasseroleslami, Hasson, Sternad (2014), which first quantifies predictability as an objective for object interactions.

Line 733: I would refrain from citing papers in un-reviewed publication outlets, like Studies in Perception and Action, which is essentially an abstract book. See also Russell & Sternad (2001) for intermittent

control or synchronization in slower oscillations and Wolpert, Miall et al (1992) Evidence for an error deadzone (1992) or Park, Marino, Charles et al (2017) Moving slowly...

Lines 745-758: Description of BS3 Control Strategy. It appears that BS3 is an inspired or "softened" form of a true bang-bang controller, in order to make it more feasible for humans to employ. More explanation of BS3 needs to be included to explain how this version is different from a true bang-bang controller with discrete on-off transitions.

Line 770: replaced 'attached to' with 'connected to'

Line 776 onwards: clumsy way of expressing the simple fact that the distance between the turning point location and the target determined what counted as a hit (+1) or overswing (-1).

Line 789-791: 'For this comparison, only the periods where the target was hit on both sides were considered, since only then was the correct energy level maintained.' What is the proportion of periods where the target was hit on both sides?

Line 798: gait and motion capture analysis is not the same category: you use motion capture to perform gait analysis .. this reads funny

Line 820: as phase lag becomes a prominent metric, some more technical information for its calculation is warranted

Statistics

How many data points per experimental configuration are entered into your analyses? The degrees of freedom in the t-tests in the results indicate that the analyses on 20 points (=20 participants). It may be useful to keep the individual trials or even the individual cycles in the analyses to represent the variance across trials.

A t-test was used to test each hypothesis. In this case, the p-values need correcting depending on the number of hypotheses tested (Tukey or Bonferroni correction). The more hypotheses are tested, the higher the type I error and the p-value needs to be lowered.

Clarify in the methods what software and what libraries/toolboxes/packages were used for data analysis and for statistical testing.

Line 837: the same distance from the ideal NNM.." same compared to what?

Line 842: what is the 'dedicated way in which the joy stick handle was moved' what does dedicated mean?

Lines 839-841: "Again, the two-sided one-sample t-test was applied to test the null hypothesis that each baseline strategy leads to a similar pendulum motion path close to the ideal NNM as the strategy applied by the participants."

Report the correlation method Pearson correlation or was it regression? In lines 179-192, ensure that all four correlations have r-values and p-values reported. Clarify whether you report a slope + p-value (Linear Regression / Linear Model) or a correlation + p-value as there are different statistical tools for determining significance of slope and correlation coefficient.
