# Supplementary material for: Finding the rhythm: Humans exploit nonlinear intrinsic dynamics of compliant systems in periodic interaction tasks
Source: PLoS Comput Biol. 2024 Sep 3;20(9):e1011478. doi: 10.1371/journal.pcbi.1011478 (PMC11398697; doi:10.1371/journal.pcbi.1011478)
Supplement: Attachment — Submitted filename: Answer_Letter.pdf [file pcbi.1011478.s004.pdf]

Annika Schmidt (an.schmidt@tum.de)
Technical University of Munich - CIT - I23
Boltzmannstr. 3
85748 Garching

PLOS Computational Biology
Academic Editor Prof. Dr. Bard Ermentrout
Section Editor Prof. Dr. Daniele Marinazzo

26th April 2024

Response to the Reviewers for the Article Submission
"Finding the rhythm: Humans exploit nonlinear intrinsic dynamics of compliant systems in periodic interaction tasks"

Dear Editors, dear Reviewers,
we are very happy that you appreciated all the effort we put into the first version of our manuscript and acknowledged that it was already a significant improvement. We are even more grateful that you have taken the time again to review our manuscript and are deeply thankful for providing us with such detailed and constructive comments. These comments have helped us to revise the manuscript once more significantly. We are confident that the latest version has substantially improved in quality, particularly in terms of presentation. Thus, large parts have been rephrased as suggested by Reviewer 2 to make our contributions as concise and as clear as possible. We address the Reviewers' comments point-by-point on the following pages. Line references refer to the new, clean version of the resubmitted manuscript. In the version with tracked changes, deleted text is again marked in red, and rewritten parts are blue.

Editors - Prof. Dr. Bard Ermentrout and Prof. Dr. Daniele Marinazzo

In light of the reviews (below this email), we would like to invite the resubmission of a significantly revised version that takes into account the reviewers' comments. Please address the major comments of Reviewer 2. Based on your reply, I will decide whether it needs to be sent out again.

We thank you for inviting us to submit a significantly revised version of our manuscript. We have taken into consideration all comments of the Reviewers, specifically addressing the major concerns of Reviewer 2. For your convenience, we here summarize the main changes:

- We have revised the overall writing style to use less complex and convoluted sentences.
- We have adjusted our definition of the *null hypothesis* and clarified our statistical methods.
- We have changed the abbreviation of the baseline strategies *BS1-3* to *BL1-3*.
- We have removed non-optimal references ([2,13] in the previous manuscript) and instead extended the reference list with the sources suggested by Reviewer 2.
- We have merged Figures 1 and 12 of the previous manuscript and carefully revised the illustrations to make the experiment setup and task better understandable.
- We have exchanged the bar plot of Figure 4 for a scatter plot to better present the correlations.
- We have improved the introduction of the analyzed metrics. As Reviewer 2 suggested, we now only use one mode metric $\eta = \eta_{man}$ to assess the distance between the participant data and the NNM.
- We have added dedicated tables (Tab. 1-3) for each pendulum configuration summarizing all metric values for the different experiment variations.
- We have revised the points of concern in the Discussion and rewrote parts of it accordingly.

We believe these changes address all Reviewers' comments to their and your satisfaction such that our revised manuscript can be considered for publication in *PLOS Computational Biology*.

Reviewer #1

The manuscript reads nicely. I think the abstract could still benefit from some work. I asked my partner, a scientist in a different field, to read the abstract, and the response was, "They lost me at the first sentence".
We thank the reviewer for acknowledging our improvements to the previous manuscript. We have revised the abstract to improve readability using feedback from scientific colleagues in different fields. We believe the abstract is now more understandable and accessible to a wider audience.

Line 72-74 and below: Please verify that you've indicated the NULL and ALTERNATIVE hypotheses right. This looks more like the alternative hypothesis to me. If I understand it, BS2 and BS3 play the role of null hypotheses. You're trying to reject these to confirm the alternative model, BS1, which is the one that involves sensitivity to the resonant frequency. If this is correct, then a simple statistical test would be to show that η_{BS1} is significantly lower than η_{BS2} and η_{BS3}. This is contingent, however, on showing that the simulations of each of the three baseline strategies used the best parameter estimates. Is this kind of optimization of simulations what is being referred to as "tuned empirically" in the "Baseline strategies ..." section, l. 706+?
We have reviewed the specification of the null hypothesis and indeed found that it was formulated incorrectly. As pointed out by the reviewer, the baseline strategies *BL2* and *BL3* (former denoted *BS2* and *BS2*) serve as null hypothesis (*H0*) that humans do not excite system resonance intuitively to fulfill the task, while *BL1* (formerly *BS1*) marks the alternative hypothesis *H1*. We have corrected these definitions throughout the text, specifically pointing them out in the Introduction (Ln. 69-73). We could not directly compare *BL1* with *BL2* and *BL3* statistically because all baseline strategies were deterministic controllers applied in simulation. These controllers were not optimized, but the amplitude was solely hand-tuned to reach the target balls. Thus, the sample size for *BL1-3* was effectively $n = 1$, which is insufficient for statistics. We hope the Reviewer understands our reasoning and agrees that the statistical comparison is clearer with the corrected definitions of the null and the alternative hypotheses.

More sophisticated statistical approaches are possible too. For example, fitting separately each of the BS1,2,3 models to the empirical data using MLE makes it possible to use the Bayesian information criterion to compare the three models, taking a number of parameters (model complexity) into consideration as well. This will simply give you the best model. I don't know if this approach is feasible here, but it would make for even stronger evidence.
During the second revision, we have considered applying more sophisticated statistical methods and have discussed the matter with expert colleagues for human user study statistics. However, we agreed that the comparison with a t-test suffices for the intended main contributions of our current research. We aimed primarily to provide data-based evidence confirming that participants indeed intuitively excited the intrinsic frequencies and motions predicted for a system's nonlinear normal mode (NNM). Additionally, comparing the participant approach with the baseline strategies indicated clear similarities with *BL1*, while the significant differences with *BL2-3* were very apparent. As a first line of research introducing NNMs and their usefulness for the analysis of human-object interactions in the community, we see the applied simple statistical methods as adequate. Nonetheless, we will aim to apply more sophisticated statistical methods in future research, which will become more relevant when analyzing the influences on human control strategies in compliant interactions in more detail.

A minor comment:Trampoline jumping and stick balancing are not exactly common everyday activities. Would bouncing a ball qualify as a valid example that is more relevant as a task of daily living?
We agree with the Reviewer that ball bouncing appears to be a more tangible everyday example task and have exchanged it for the swing-stick example (Line 11).

Reviewer #2

The authors provided an extensive revision of their manuscript in response to our comments. The re-writing of the manuscript has significantly improved readability. The authors have further strengthened some of their arguments by adding references and clarifying metrics, figures, and analyses. While this revised manuscript is a significant step forward, there is still a long list of comments and suggestions to further improve the manuscript. While the list is long, the study is scientifically valuable and, with more work, will hopefully become more rigorous in its presentation.
We thank that Reviewer for viewing our research as scientifically valuable and acknowledging the significant improvements made to the previous manuscript. We appreciate that the Reviewer took extended time and effort to review our work in so much detail and provide us with very constructive comments for improvements. We believe that addressing them has further improved the presentation of our work substantially and are confident that our manuscript now meets the high standards of the journal *PLOS Computational Biology*.

Major Comments

1. While the writing has improved, many passages, especially in the discussion, are still quite wordy and 'unconstrained'. Tightening the language would improve readability. Some choices of words and phrases still indicate that a native speaker should go over the text.
   While revising the manuscript, we have paid special attention to keeping sentences short and 'constrained'. We have checked the grammar and writing with multiple tools and had two native speakers go through the text.

2. The results are inconsistently reported with seemingly select statistics, and the results of Experiment 2 are only reported visually. The human data obtained in the three experiments should be consistently compared with the three hypothesized strategies. The authors have introduced three metrics: task success, mode metric, and phase lag. For each experiment, each of these metrics can be compared with the three hypothesized strategies. Coupled with the result that, for example, the mode metric was lowest for BS1, it can be inferred that participant trajectories were closest to the ideal NNM.
   As suggested by the Reviewer, we have clarified the four metrics we investigated: 1) task success based on hit scores, 2) each system's oscillation frequency f_{osc}, 3) the mode metric η evaluating the distance from the NNM and 4) the phase lag ϕ and associated deflection ratio ρ between the motor link as input and pendulum link as output. We have added Tables 1-3, one for each pendulum configuration, where all metrics for the different experiment variations are reported, including the sample sizes n. We thank the Reviewer for the suggestion, as we believe the more consistent presentation of the metrics in table form improves the readability of our manuscript.

3. The experimental task is still unclear and difficult to understand, especially as different aspects of it are shown in three different figures. While the task variations are clear (reduced target size, etc.), the way the task is carried out by a human remains hard to visualize. For example, did the subject move their arm laterally to move the apparatus, or did they rotate the wrist, and in what direction? Provide more description on exactly how the user manipulated the apparatus. Combining at least the first and second figure will help.
   As suggested by the Reviewer, we have combined Figure 1 and Figure 12 of the previous manuscript in Figure 1 of the revised version. We have spent extended time adjusting the perspectives to properly visualize the movement direction of the handle during the experiments and point out the setup and positioning of the participants relative to the screen. We also now show the three investigated double pendulum configurations individually. We believe that our changes make the experiment arrangement and task clear now.

4. The discussion of the control strategies is sometimes contradictory. The frequencies by themselves are not sufficient indicators of whether participants perform feedback (reactive) or feedforward control (prospective) as the authors also argue that participants could be modulating their arm stiffness to shift the NNM mode to lower frequencies. The more relevant difference between BS1 and BS2 is that BS1 requires low forces (low input, thus resonance and using NNM), and BS2 requires greater forces (large input, not exploiting resonance and not using NNM). It may be beneficial to have a different name for BS2. In Experiment 2, the authors argue that participants shifted the oscillation frequency farther away from BS1 to become more accurate. Does this not negate the hypothesis of the authors? If participants want to be more accurate, they should more accurately match their oscillation frequency to the "resonant" frequency of the system.
   We acknowledge the concerns raised by the Reviewer and have weakened the interpreted relations to *reactive* and *prospective/ anticipatory* control approaches throughout the manuscript. However, we want to point out that *BL1* (formerly *BS1*) is the strategy that requires larger forces (despite lower motor link deflections) as the spring forces are higher when the deflection difference of the motor link and upper pendulum link is large. Instead, *BL2* (formerly *BL2*) only causes little spring force as the motor link and the upper pendulum link almost move synchronously, leading to lower and smoother force curves. This is in line with previous research that participants are willing to sacrifice the smoothness of forces and trajectories when instead applying a control approach that lets them better predict the system dynamics, i.e., exciting the resonance with *BL1*. We had chosen to declare *BL2* to be more *reactive* to refer to the fact that humans were more visually dependent on the motion of the pendulum as they were not exploiting the spring and thus only felt little forces. However, we see how this can be confusing and refrain from this declaration in the revised manuscript. Accordingly, we revised the motivation and references for *BL2*, emphasizing the expected smoother and lower force curve of this approach (Ln. 74-79, Ln. 728-744).
   Concerning the Reviewer's comment about the findings of Experiment 2, we agree that, based on theory, it could have been expected that participants were closer to the ideal eigenfrequency to be more accurate. We address this in the Discussion in Line 436. However, we also point out that the human might not be aware that better matching the eigendynamics of the pendulum will make them more accurate. The increased precision requirement due to smaller targets might have caused participants to intuitively want to slow down the system, possibly related to the limited information bandwidth defined by Fitt's Law. To accomplish this, they might have softened their wrist stiffness, which is now addressed in the revised Discussion in Lines 438-442.

5. The discussion section talks about aspects of the data that were not reported in the results. The sinusoidal shape on the human trajectory is in line with the BS1 strategy and the initial explorative movements that were not shown in the results. These data should either be shown or eliminate reference to these data aspects in the discussion
   The part of the Discussion in the previous manuscript that addresses the commanded input shape to the motor link (Ln. 299-308 in the previous version) was mainly aimed to emphasize that humans appear not only sensitive to the required oscillation frequency but also to the correct signal shape that can excite it. The dedicated shape of this signal was of secondary interest, although it appeared most similar to a sine wave in the conducted experiments when averaged over all participants. In the referred section, we wanted to point out that in an interaction with a different compliant object, the needed input shape could look different, but we still suspect that the human could successfully excite intrinsic oscillations. However, based on the Reviewer's comment, we see how this paragraph was confusing and might have put too much emphasis on a vague expectation. Thus, we have majorly shortened the considered paragraph, only pointing out the observations of the current experiment (Ln. 320-322). Likewise, we only refer to the unrecorded data from the familiarization period to justify why learning effects did not have to be considered in the data analysis (Ln. 343-347).

6. The target hit score seems somewhat biased. It is argued that overswing would alter the energy level and, therefore would confound the comparison with the NNM. Do undershoots or missed targets not lead to a lower energy mode? If so, should they not also count as -1 as the overswings? A score report that splits between hits and misses, both over and undershoots, may help clarify this. A different count of the hits may also reveal different patterns in the correlations with the phase lag
The interpretations of the Reviewer about the alterations of the energy level are correct. Overshoots and undershoots of the targets alike indicated that the participants did not match the required energy level. Both errors did not influence the hit scores in our experiments. However, it seems like our presentation was not clear in this regard. We acknowledge that the assignment of *hits*$= 1$, *undershoot*$= 0$ (formerly *no reach*), *overshoot*$= -1$ (formerly *overswing*) in Line 780 of the previous manuscript might have been confusing. The value assignments were based on the implementation of the hit count. Collision detection of the lower pendulum link mesh and a target ball mesh triggered that a point was added to the hit score. If the link completely swang through the target, this shortly added point was again subtracted, thus denoting the *overshoot* as -1. If the target was not reached, i.e., *undershoot*, no point was added nor subtracted, thus the denotation of 0. Hence, neither *undershoot* nor *overshoot* of the target affected the hit score, meaning that the "penalty" of injecting too much or too little energy into the pendulum system was identical in the experiments. We have clarified that the assignment of the numbers categorizing the hit attempts was not influencing the score but was mainly important in the analysis to identify which error occurred (Ln. 776-780). Additionally, we have added the number of *overshoots* and *undershoots* to Table 1-3 as suggested by the Reviewer.

7. The two mode metrics seem to obtain different results when compared with the NNMs. This is confusing. It appears that the mode metric of the full state space is the more complete one, hence the one that should be reported. While some differences may be less favorable for the hypothesized strategy, I assume the new suggested statistical comparison will render new and likely supportive results for BS1.
We took the Reviewer's comment into consideration and have decided to follow the recommendation to only use one mode metric $\eta = \eta_{man}$. As correctly pointed out by the Reviewer, the distance over the full state is more complete. The reason for also reporting the distance in position space only was that we believed it to be better imaginable for the reader as a 4D-space comparison is hard to visualize. However, we now agree that it was confusing, and by visualizing the distances in position and velocity space individually, the concept should still be graspable. Thus, we have streamlined the manuscript to include only one mode metric, which is reported in Table 1-3 for all pendulum configurations and experiment variations for a better overview.

8. Overall, the concept of energy levels must be discussed in more detail. It is still unclear to me how different energy levels are achieved/excited. Is it by adjusting the initial angles of the pendulum or by introducing a non-zero velocity as an initial condition or?
We understand that the concept of NNM is not intuitive to grasp. We have spent dedicated time to introduce the concept and methods to derive the NNMs of a double pendulum in the method section (Line 480), showcasing the evolution of period times and brake mode trajectories for different energy levels. For the conducted user study, only one specific energy level was defined, which was merely important to determine the location of the targets and the explicit brake orbit trajectory of the NNM taken as reference. Thus, the concept of different energy levels and how they influence the NNM progression was only of minor relevance for the experiments. More importantly, it should be conveyed that it is possible to predict the specific trajectory of nonlinear systems (for different energy levels). Although we agree that we could further detail the theoretical background and evolution of NNM for different energy levels, we believe it might provide too much information such that it could distract from the relevant aspects and confuse the reader. However, we have

provided multiple sources ([26-29, 54] in the current manuscript) that give more detail on the theory, computation, and application of NNM at different energy levels. We hope the Reviewer understands our reasoning for not detailing the NNM energy levels in even more detail in the submitted manuscript.

9. The simulations for BS2 and BS3 should be explained more prominently as they serve as reference or alternative hypothesis throughout the paper.
   As suggested by the Reviewer, we extended the information in the introduction about *BL2-3* and adjusted the cited references to better motivate the choice of these alternatives (Ln. 71-84). We believe this sufficiently introduces and motivates our choice of taking *BL2-3* as possible baseline strategies that could underlie the human control approach.

We hope our answers and adjustments to the manuscript sufficiently address the major concerns of Reviewer 2. We have also taken into account all detailed comments. The individual answers are upcoming in the following. Whenever possible, we have grouped comments to provide a common answer.

Detailed Comments

1. **Line 5** onwards: I appreciate the authors to better situate their work in existent understanding of human motor control. As I am keenly interested in this area of study, I probed into the cited papers. Unfortunately, I found some of the new references non-optimal. For example, amongst the references for predicting and following chaotic behavior, reference 2 is a non-refereed 4-page extended abstract. A similar extended abstract is cited later. For a journal like PLoS Comp Biology, it may be better to use peer-reviewed papers, especially in the introduction. The paper by Goodman et al (ref 9) seems more to the point here. Ref 5 is not really on resonance, may be better Kugler and Turvey (1987) and Raftery, Cusumano, & Sternad (2008). I also do not think that reference 7 is a good reference for resonance and dynamic stability
   Line 16: This point was also made by Maurice, Hogan, Sternad (2018).
   Line 20: Reference 20 is again an unrefereed extended abstract, in the current context, 'prospective control' appears misleading as it was only an experimental manipulation, not a reliable assessment or simulation of prospective control. See also Sharif Razavian, Sadeghi, et al (2023) for this point.
   Line 26: See also Nasseroleslami, Hasson, Sternad (2014) for the comparison between predictability, stability and smoothness.
   We thank the reviewer for pointing out the inadequate references ([2,13] in the previous manuscript). We have replaced these references with peer-reviewed papers and extended the additional references suggested by the Reviewer ([2,4,8,13,14,16,22,31,32,33,45] in the new manuscript). Additionally, we have revised the Discussion to address the points raised by the Reviewer, which made some of the previous references obsolete ([5,17,26,28,41,42,43,45,61] in the previous manuscript). Furthermore, we have overall weakened the suspected implications of *prospective control*, only mentioning it in the introduction. Here, we have chosen to rename it to *anticipatory control* as discussions with colleagues revealed that this term appeared less confusing. We believe these changes should address the Reviewer's concerns to their satisfaction.

2. **Line 67, Figure 1**: I fail to see the blue, orange and yellow configuration in the figure? Only the targets are colored and the pendulum links are white. Why not simply showing three pendulums separately? Also make the text follow the figure: first explain the left panel. Also, is the hand orientation really matched with the hand figure? Then, the hand would overlap or 'collide' with the double pendulum. Would it not make more sense to have the hand coming from the right to

hold the red handle? Unnecessary: the hand looks as if it is manipulated by the left hand. Are the indicator panels for the remaining time and target hit score really as large? Figure 12 may be combined with Figure 1 to not distribute different illustrations of the experiment throughout the paper.
We have carefully revised Figure 1 and merged it with Figure 12 from the previous manuscript as suggested. We also followed the Reviewer's advice to show the tested pendulum configurations in separate sub-figures (Fig. 1c). We have paid special attention to making the participant's orientation during the experiment and their hand motions clearer by replacing the previous photograph with a better picture from a different perspective and pairing it with an analogous sketch. We have asked multiple colleagues who were not involved in the experiment for their feedback to ensure that the figure is now better understandable to uninformed readers.

3. **Line 75**: I would not call the strategies 'baseline strategies', later abbreviated to BS. First, the acronym BS has negative connotations in English and American slang. I suggest to call them hypothesized strategy for the resonance tuning one and alternative strategies for the other two. Or just short names. I also have reservations about strategy 2, as explained in the general comment above.
We have incorporated the Reviewer's suggestion to change the abbreviations of the baseline strategies from *BS* to *BL* to avoid negative connotations. However, we have refrained from giving different names to *BL2* and *BL3*, as all considered strategies were based on control approaches that have been observed in humans in different research works. Although we hypothesized that the approach for the given experiment task should be most similar to *BL1*, we viewed all compared control approaches as valid and possible strategies.

4. **Line 81**: The two metrics are not a contrast, delete 'on one hand' and 'other hand'.
We have rephrased the Introduction to better introduce the metrics used for comparison and have incorporated the Reviewer's advice here (Line 85-103).

5. **Line 105**: It may help readability to summarize the experimental task for the subjects, including the duration and scoring achieved. Also, state the exact conditions on Exp 1, 2, 3. It would also help to have a brief summary of the simulated strategies, BS1, 2, 3.
We incorporated the Reviewer's suggestion by extending the motivation for the baseline strategies (Ln. 63-84) and clearly explaining the analyzed metrics (Ln. 84-102) in the Introduction. Additionally, we improved Figure 1 and added a short section at the beginning of the Results (Line 105) summarizing the experiment setup and variations. Combined, we believe the exact conditions of the experiments are now clarified.

6. **Line 112**: It is typically useful to first state how participants achieved the task. In this case, the explicit instruction was to hit targets. Hence reporting target scores would be good. May be also list overswings and hits separately so that the reader gets an overall idea how difficult or easy the task was. I also would not refer to the resonance strategy as null hypothesis, because null hypothesis denotes the claim that the effect being studied does not exist or when there is no relationship between two conditions.
We have addressed the Reviewer's comment and have changed the order in which the individual metrics are presented. As suggested, we now report the **Task success** (Line 134) with the hit scores first. Additionally, we added the numbers for *overshoots* (formerly *overswings*) and *undershoots* (formerly *no reach*) for each pendulum configuration and every experiment in the Tables 1-3. Further, we have reformulated the hypotheses statement, as indeed *BL2* and *BL3* served as null hypothesis, while *BL1* denoted the alternative hypothesis (Ln. 68-73).

7. **Line 116**: Which statistical comparison was applied? A linear mixed model with the two oscillations or did you use t-tests for each of the two comparisons? If yes, them report t statistics with degrees

of freedom and Bonferroni adjustments. See comment on statistics. Table 1: use the same labels for the pendulums.

Line 131: Which data were entered to calculate the means and standard deviations? All trials and all subjects? Again, report the statistical results.

As correctly pointed out, we have used the t-test for the statistical comparison. We have clarified the methods and emphasized that we appended the three best trials per participant to one combined trial used for the statistics (Line 840). The respective sample size n for each experiment is now immediately apparent from Tables 1-3. These tables also include the means and standard deviations for all metrics. We added the missing t-statistics for the analysis of the oscillation frequency in Line 153 and the mode metric in Line 174-175 as requested by the Reviewer.

Furthermore, it is specifically emphasized now why a Bonferroni p-value adjustment was regarded expedient in our specific case (Ln. 857-863. For our comparison with the baseline strategies, lowering the α value according to Bonferroni would have uncommonly worked in favor of our hypothesis that there exists no significant difference to the expected eigenfrequency or the control strategy *BL1*. Nevertheless, we now address the effect of adjusting the α-level to $\alpha = \alpha_{5\%}/3_{BL} = 1.7\%$ to test the null hypothesis that the participant data was significantly different from *BL2-3*. Since these comparisons showed strong significance ($p < .001$), the results remain identical even after the p-value adjustment.

8. Why were two mode metrics calculated, one for position and one for the full state space? The results are very similar and only one of the two is needed. I suggest the more complete metric for the full state space.

   As already addressed in the major concern **#7**, we agree with the suggestion of the Reviewer and now only use one mode metric η, which was η_{man} in the previous manuscript version.

9. **Figure 2** does not have red traces?

   The red traces in the legend of Figure 2 were meant to refer to the targets. We have clarified this by changing it to a circle shape in the new version of the manuscript.

10. **Line 139-146**: The statistical evaluations for these important tests are obscure, and the conclusion that there is no significant difference, although P90 does differ, is sketchy. There are two significant p-values, which suggest that participants' dynamics are somehow different from BS1 (in position for P0 and over the full manifold for P90). While participants are closer to BS1 than to the other strategies, they still differ when using this analysis. Suggestion: compare the subjects' data (include 3 trials as separate estimates) using a linear mixed model with the 3 hypothesized strategies. The mode metric is likely to be lowest for BS1 as hypothesized.

    Table 1: Comparing values of BS1 and Exp1, it seems that participants were able to perform the task "better" than BS1 based on the metrics introduced. Could this suggest that even BS1 is not the correct baseline strategy to compare against the participant strategy? I think it is important for the authors to touch on this in the discussion.

    We acknowledge the Reviewer's concern and agree that the reporting was confusing, especially when considering both mode metrics. We believe since we now only include one mode metric $\eta = \eta_{man}$, the results are less obscure. Nevertheless, we obtain no significant difference for the *P0* configuration but see significance when comparing η for *P90*. Although we agree with the Reviewer's comment that more sophisticated statistical methods could be applied, we see the simple t-test as sufficient for the scope of the presented research. However, we have now specifically addressed the possible implications of the significant differences that were found for the mode metric in the *P90* configuration in the Results section (Ln. 174-176) and point out in the Discussion that participants might apply a more sophisticated control signal than the simple sine-wave commanded by *BL1* (Ln. 318-320). We plan to do a more in-depth comparison of the human control strategy and *BL1* with more varied pendulum configurations and sophisticated statistics in future research.

11. **Figure 2**: target locations are ellipses indicating different axes. Better make axes the same +1 to -1 to give a more realistic impression. May be the same for joint space depiction.
    The plot ratio has been adjusted to give a more realistic impression.

12. The section on applied handle motion is hard to follow as its rationale was not introduced or prepared in any way. See more detailed comments in the following:
    We have revised the Introduction to give a clearer motivation as to why and how the handle motion was analyzed (Ln. 99-102). We have also revised the Results section of the handle motion (Ln. 181f) and believe it is easier to follow now.

13. **Line 155-157**: Sentence 'Therefore ... require a control input' is unclear. I understand that if the handle is displaced, the conservative system would continue with the handle moving accordingly. Would this not imply that the larger handle deflection and sinusoidal motion with the initial amplitude needs to continue? Alternatively, I understand that at resonance, the input amplitude can be small. In this case, the friction in the pendulum requires continued input. These three observations do not quite converge.
    It appears that our explanations in the previous manuscript version were confusing. To clarify, in the conservative case, where no friction is present, the handle always remains static at $\theta = 0$. When the pendulum links are deflected from the correct initial positions, the system will continuously move along the computed NNM without requiring a control input. The handle should not be moved. It is possible to model the frictionless system for participant interactions. However, this would neither enable a realistic object interaction nor allow an investigation of how humans excite compliant nonlinear systems. In an ideally conservative system, participants would solely be required to hold the handle statically for the duration of the experiment, as injecting additional energy will mostly destabilize the system. To investigate how humans excite and stabilize oscillating nonlinear systems, friction had to be added to the virtual system. In this case, the human had to continuously inject energy into the system to sustain the oscillation at a constant amplitude to hit the targets. This energy injection was realized by moving the motor link, which was never required in the ideal conservative case. However, although the motor link needs to be moved in the friction case to inject energy, it was expected that its amplitude would remain small compared to the pendulum link oscillation when following the concept of resonance, leading to output amplification. This was the reason to compare the motor link deflection as system input and the pendulum oscillation as output. The idea behind this concept is now stated in the Introduction (Ln. 99-102) and has been rephrased in the pointed-out paragraph in the new version of the manuscript (Ln. 184-187). We thank the Reviewer for pointing out the confusing wording.

14. **Line 160:** If 1.55 rad is the amplitude, then it would be better to say that the amplitude of P0 was 1.5 rad, not moved more ... I do not see the numbers how this amounts to 14% or 17% larger amplitude. Is there an explanation for the offset of the human strategy compared to the simulated strategy? Also, these values are 0.5pi, as stated below. Relate this number to .5pi before when the numbers are presented.
    It appears that the presentation of our results was again confusing. In fact, 1.55 rad was not the pendulum link amplitude in the *P0* configuration, but the deflection ratio between the maximum deflection of the motor and the link over one period, i.e., $\rho = \texttt{max}(\theta)/\texttt{max}(q_1)$. We now introduce the relevant variable ρ already in the Introduction (Line 100). When presenting the results for this metric, we now first mention the observed deflections of q_1 and θ individually (Ln. 189-192) and then set them into relation by determining the quotient between the two values. We have also added this value for all pendulum configurations and experiment variations in Tables 1-3 and clarify here again that $\rho = \frac{\max(\theta)}{\max(q_1)}$. Analogously, we now explain the meaning of the $\pi/2$ phase lag value in the Introduction (Line 99). We believe these changes clarify the carried-out analysis and the reasoning behind it. We thank the Reviewer for raising this point.

15. **Line 179:** past tense is confusing here, the phase will be compared to task success (task success should be mentioned first in the results).
We agree with the Reviewer and have changed the order of the reported metrics and their results as suggested.

16. **Lines 179-192:** correlations were illustrated in Figure 6 with participants ranked by either the two score metrics with a linear trend line, paired with values of the phase lag highlighted by a separate linear trend line. This is a confusing way to report correlation. Illustrate correlation analysis via scatter plots of X vs. Y. Consider also adding confidence bands.
According to the Reviewer's suggestion, we have replotted Figure 4, using a scatter plot now instead of the previous bar plot. However, we have not changed the x- and y-axis, but we believe, nonetheless, that the new version is more intuitively understandable when talking about correlation comparisons. We thank the Reviewer for their comment as we believe it further improves the presentation of our results.

17. **Line 188:** both correlation coefficient and p-value are relatively far from significance, rephrase.
Line 192: the highest value was reported to be .74pi?
We thank the Reviewer for pointing out the confusing phrasing in the pointed-out paragraph. We have rephrased the result presentation about the correlation and corrected the wrongly stated maximum phase lag value (Ln. 216-222).

18. **Line 207:** replace 'stretched' pendulum with 'extended' pendulum here and below. Do the reported hit numbers for the participants include both hits and overswing? Please report task success with all three occurrences.
We have exchanged the words as suggested by the Reviewer and now report the *hits*, *underschoots* and *overshoots* separately for all pendulum configurations and experiments in Tables 1-3.

19. **Lines 217-220:** If I understand this correctly, in order to become more accurate, participants shifted the oscillation frequency farther away from BS1? Does this not negate the hypothesis of the authors? If participants want to be more accurate, they should more accurately match their oscillation frequency to the "resonant" frequency of the system. Also, the tests should again be corrected and include 3 trials.
We have considered the points raised by the Reviewer and have revised the Discussion to address this point (Ln. 436-444). We now specifically mention the observation that the performance decrease in matching the eigenfrequency when being more accurate might be counter-intuitive based on the theory. However, we believe participants might have intuitively preferred the slower system oscillation for increased task difficulty due to the limited information bandwidth of humans described by Fitt's Law, thus sub-consciously adjusting their wrist stiffness to slow down the system. We thank the Reviewer for raising this point.

20. **Figures 5, 6 and 7**: Axis labels saying position or velocity are redundant. Could be removed to reduce clutter. Also, for the two left panels, y axis could be θ [rad]. What denotes the green color in Figure 5?
Caption word over all correct to 'overall'. Report hit rate consistently as number, not percent.
We have adjusted the figures as suggested by the Reviewer.

21. **Line 245:** "..a setup is unlikely, 'such that' we used an additional..". 'such that' is not English here
The sentence has been rephrased, and the grammar of the complete manuscript has been carefully reviewed by a native English speaker.

22. **Line 275:** "..data supporting the 'intuitive' hypothesis.." The hypotheses tested here are not purely intuitive but theoretically and quantitatively motivated.
We have rephrased the relevant sentence (Line 294).

23. The results of Experiment 2 also warrant a quantitative comparison with the mode metric. As it stands, the results are only reported visually.
Lines 250-270: the results of this experimental condition should again be compared to the three hypothesized strategies, using the three descriptive metrics: target hits, mode metric, and phase lag.
As suggested by the Reviewer, we have added dedicated Tables 1-3 that summarize all metrics for all tested pendulum configurations in all experiment variations.

24. **Line 304:** the fact that human handle trajectories were close to sinusoidal, similar to the sinusoidal input of BS1 was not really dealt with and tested in the results. If this is a strong observation, this should be tested.
Overlaying the driven handle trajectory averaged over all participants, and the one commanded by *BL1* in Figure 3 (left) showed a very apparent shape resemblance. This was the reason to suggest that the overall participant control strategy might be similar to the sinusoidal control trajectory of *BL1*. However, we agree with the Reviewer that this implication derived from the given results might have been too strong, as the individual handle trajectories per participant show a wider signal shape variety, and the similarity with a sine wave was not analyzed in further detail. Thus, we now focus mainly on the comparison of the phase lags and deflection ratios in the Results section. As already explained in the answer to the major concern **#5**, the referenced lines in the Discussion were intended to point out that humans might be not only sensitive to intrinsic oscillation frequencies but also to different control input shapes that can excite them. In the conducted experiment, *BL1* showcases that a simple sine-wave would have been sufficient to excite resonance in the system, but the data suggests that the human strategy might be more sophisticated, indicated by the significant difference in the *P90* configuration. The variation between the participants also suggests that the applied control strategy might depend on individual preferences and skills. The relevant paragraph in the Discussion has been rephrased accordingly (Ln. 320-322).

25. **Lines 310-322:** In the rest of the manuscript, the "training" period was completely omitted in the analysis. Yet, data from that period are discussed here as though the reader has been previously presented some of the trends and behaviors discussed. For example, on line 318 it is mentioned "the learning curve during the training period appeared very steep". If this is an important point that needs to be discussed, then perhaps a figure should be added to show the mentioned learning curve. The way the text stands now, it feels very vague and unsubstantiated.
We agree with the Reviewer's comment that we should not discuss data that was not previously analyzed and reported. The main reason for mentioning participant behavior during the training time in the Discussion was to justify why the inclusion of learning effects over the separate experiment trials was regarded as unnecessary in our experiment setup. We regard this explanation as essential for the reader to understand our reasoning to exclude learning and have thus left it in the manuscript (Ln. 343-347). However, we have rephrased the paragraph to only emphasize our motivations and do not further refer to this data anywhere else.

26. **Line 327:** The statement that subjects adopted a prospective control approach appears bold as it is only based on the assumption that resonance behavior is more predictable, as reported in another study. Such relations need to be reported more carefully as conjectures rather than facts.
We agree with the Reviewer's observations that the implications for prospective/ anticipatory control based on our experiment results might have been too strong. We now only introduce the principle of prospective/anticipatory control in the Introduction (Ln. 15-16). Otherwise, we only point out that better predictability might be a reason for the high resonance sensitivity of humans (Ln. 329-336) but do not draw further conclusions about the underlying (biological) control strategy.

27. **Lines 310-342:** this is quite lengthy and could be made more succinct.
As suggested, we have revised the mentioned paragraph and condensed it, now only spanning Lines 323-336.

28. **Line 348:** Could it be that the close location of the handle on the screen biased the subject as otherwise there may be an overlap?
Unfortunately, we are unsure what the Reviewer is referring to here. We are happy to address the Reviewer's concern, if they could provide us with more context.

29. **Line 359:** rephrase 'dedicated'
The sentence has been rephrased accordingly (Ln. 365-366).

30. **Line 365:** this discussion is confusing: it is proposed that the visual display biased subjects and that this then causes deviations from the sinusoidal trajectory shape. This contradicts what was said above.
We acknowledge that the phrasing of our results appeared confusing in this context. In fact, we did not want to convey that the shape of the trajectory, being sinusoidal or otherwise, was influenced by the visual display. Solely the observed position offset of the mean handle position away from zero was hypothesized to stem from visual influences. In the *P0* configuration (Fig. 6a, left), the handle oscillation was equally distributed around the defined equilibrium position $q_{eq_1} = 0$ illustrated in Figure 8a-c by a dotted line. In contrast, evaluating the oscillation of the handle for the *P90* configuration (Fig. 6b, left), all participants (independent of the specific shape of the θ curve) appeared to move around a shifted equilibrium position of $-7.44°$ (Line 201). This observation was seen as **unrelated** to the overall shape of the handle curve and possible similarities with a sinusoidal trajectory. We have reformulated the corresponding part in the Discussion (Ln. 357-365), independent of discussing the θ-signal shape to avoid confusion. We apologize for the confusing reporting of our results and thank the Reviewer for the valuable feedback to improve the presentation of our findings.

31. **Line 398-411:** these statements about chaotic motion and control are confusing. First, the human data were not analyzed whether they revealed chaotic features. They were only compared to the NNM. The discussion about chaotic control or predicting chaotic evolution appear disconnected from the results.
We agree with the Reviewer that the stated details in this paragraph were not directly connected to our research and have chosen to remove them from the Discussion.

32. Line **413-432:** I am not convinced by the argument made in this section explaining why participants slightly reduced their movement frequency when the target size was reduced. Fitt's Law does not seem like a valid argument to be made here. While it is true that humans often obey the speed-accuracy trade-off, this is not always the case as conflicting constraints may suppress it: (Kelso, Southard, Goodman, 1979). But if speed-accuracy trade-off is the primary driving factor, then BS2 must be the best strategy as it is the slowest, hence the most accurate strategy.
As already addressed in the answer to the detailed comment **#19** in this letter, the Reviewer raised a relevant question. We agree that solely based on theoretical considerations, it could be expected that the participants would better match the eigenfrequency to increase accuracy. This is now specifically pointed out in the manuscript (Line 436). Nevertheless, we still consider that the lowering of the oscillation frequency could be related to Fitt's Law, but we revised our reasoning. We now argue that humans might have intentionally, although subconsciously, reduced the oscillation frequency slightly by decreasing their wrist stiffness and recalculating the NNM for this case (Ln. 428-434). We thank the Reviewer for pointing this out, as we believe it has improved the interpretation of our experiment results.

33. **Line 457:** sentence grammar 'it is not only ..
We have overall revised the manuscript's grammar and corrected the mentioned sentence (Line 459).

34. **Line 460:** Here resonance sensitivity and prospective control is equated. This is not correct.
As mentioned in answers to previous points, we have refrained from drawing further implications of prospective/ anticipatory control, such that this sentence and its implications are no longer included in the manuscript.

35. **Figure 8a:** increase the turning points (hollow dots) that are hardly visible to ease understanding of the figure. **Figure caption:** delete 'a' in line 2, change second d) to e)
Also, it is nearly impossible to see these dotted lines on the figure, if they are important to be pointed out, then perhaps use a different color to plot them.
We have increased the hollow dots of the turning points as suggested and corrected the caption. We also made sure to improve the visibility of the dotted line.

36. **Line 541:** exemplary, . . . not English, change to: As an example,..
We have rephrased the sentence as suggested (Line 544).

37. **Figure 9, caption:** joint space is depicted on the right, not left
We thank the Reviewer for pointing out this mix-up and have corrected the caption of Figure 9.

38. **Line 561:** "level E =2.5 J that is arbitrarily chosen". In the previous version of the manuscript, the justification for this choice of energy level was based on "human preference". Knowing that, to say a value was arbitrarily chosen seems like the authors did not want to spend an extra sentence giving a deeper and more satisfying justification for this choice.
We have carefully read through our first initial manuscript version, which can also still be found on *bioRxiv*. Here, we stated in Line 169, "Since the mode changes its shape with energy, for both configurations, a fixed energy level of 2.5 J was set for the investigation in the human user study." Unfortunately, we cannot derive to which part the Reviewer refers when saying that we initially stated the energy level was based on "human preference". While we did not base the energy level on any human preference, we did state in the initial (Line 659) and the previous (Line 558) versions of the manuscript that "The set parameter values of the [pendulum] systems were loosely based on the dimensions of a human arm". However, these parameter settings only referred to the pendulum link length l_1 and l_2 and their masses m_1 and m_2 and were just taken for very rough dimensioning. This was not directly related to the energy level. In the current manuscript, the same sentence can be found in Line 560. We apologize that we seem to not have presented our methods clearly in the initial version, but we believe this has majorly improved in the latest version.

39. **Lines 561-562:** "In this investigation, only the first, more stable mode will be considered". How was 'more stable' quantified and identified?
Again, the Reviewer raises a valid question. The stability of a mode can be derived from the progression of the characteristic multipliers, i.e., the eigenvalues of the Poincaré map. The detailed explanation of how this can be determined would not fit in the scope of the paper, but we have added this specific information in the manuscript (Line 562) with a reference to related research of our group, where more details about this can be found [54].

40. **Line 581:** "two links with ball geometry were added as targets that had to be reached." I do not understand whether the ball geometry refers to the links or the targets, rephrase.
We see how the phrasing might be confusing to a reader that is not familiar with the software *Gazebo*, where every body that is part of a model is referred to as a *link*. To avoid this confusion, we have rephrased the relevant sentence and do not refer to the target balls as *link* anymore (Line 582).

41. **Line 583:** This is not a collision with force, change collision to the link reached or hit the target.
In the new version of the manuscript, we have clarified that only mesh collisions were detected, and no physical collisions leading to interaction forces were defined between any of the links. Thus, the pendulum could freely move through the target balls, and the upper pendulum links could pass through the motor link (Ln. 585-587).

42. **Lines 598-600:** "In the case of the random control, the amplitude A and frequency ω were pseudo-randomly resampled every 0.1 s with $A \sim \mathcal{U}(1, 10)$ and $\omega \sim \mathcal{U}(0, 10)$ rad s^{-1}.". For $A \sim \mathcal{U}(1, 10)$, does this mean that the amplitude of oscillation could be 10 radians? Which means multiple complete rotations (2π) of the link?
The Reviewer's observation is correct. If given more time before the next resample, the motor link would have been commanded to carry out more than one complete rotation $A = 2\pi = 6.28$. However, since the time before the amplitude changed was very short, this was never attempted. We see it as a valid comment that we could have limited the resampling to $A \approx U$ (1, 6.28). Nevertheless, the main point of this experiment was to show the reachable space of the pendulum systems and prove that they do not always follow the NNM. Choosing a smaller maximum value would not alter the results of this experiment.

43. **Figure 12:** While these figures now make understanding the task easier, I wonder what the hand motion was: wrist flexion and extension in the general position shown in Figure 12c? Or was it a forward/backward movement? How did this match with the visual display of the red handle? The experiment figure would be better combined with the initial figure in the introduction.
As suggested, we have merged Figure 12 with Figure 1 and no longer include the images of Figure 12 separately in the method section.

44. **Line 675:** "this specific parameter" there is no parameter value mentioned yet. I suppose you mean the value mentioned in the next sentence? Also, the sentence does not give a real rationale: if humans add their own stiffness, why do you need a stiffness in the virtual motor link?
We apologize for the unclear phrasing. As suspected by the Reviewer, we were referring to the upper spring stiffness k_1. We have now rewritten this sentence to avoid confusion (Line 675). Regarding the second part of the question: it was a specific choice to study the introduced double pendulum system (Fig. 8) to model the feeling of holding a flexible stick. The human grabs the joystick corresponding to the red handle on the screen and feels the spring forces that the pendulum links apply (Fig. 1a). Instead, the Reviewer suggests *another* system, where the red link would be removed, and the human directly holds the upper pendulum link, only feeling the forces from the connected lower link. While this model can also be modeled, we simply chose a different one for the conducted study, where k_1 acts in series to the human arm stiffness. Thus, the cumulative stiffness affecting the upper pendulum link was a parameter participants could have (subconsciously) influenced. To understand this possible influence, we were interested if and how upper spring stiffness changes affected the system dynamics and the shape of the NNM. We are aware that this view is simplified and does not account for the high complexity of the human neuromuscular system.

45. Line 704-705: "After one completed experiment, the participants filled out a NASA TLX-inspired questionnaire." Why include this if the questionnaire is not discussed anywhere else in the manuscript?
We agree with the Reviewer that it is irrelevant to mention the NASA TLX-inspired questionnaire if no results from it are reported. Why it has helped us to better interpret the participant results and confirm the correct execution of the setup and experiment, no additional findings that contribute to our research could be derived. Thus, we now refrain from mentioning the questionnaire at all in the manuscript.

46. **Line 706**: I would rephrase baseline strategies to hypothesized strategy and alternative strategies, HS and AS1 and AS2. Or give is short names. To label strategies BS is too close to slang for 'bull shit' (sorry).
As already addressed in the answer to the detailed comment **#3** of this letter, we have changed the abbreviations for baseline strategy to *BL1-3*. However, we have refrained from giving a distinctively different name to *BL1* compared to *BL2-3*, as we did view all three strategies as valid approaches that the participants could have applied.

47. **Line 725:** a reference for 'humans seek predictability' is Nasseroleslami, Hasson, Sternad (2014), which first quantifies predictability as an objective for object interactions.
We have added the suggested source [16].

48. **Line 733:** I would refrain from citing papers in un-reviewed publication outlets, like Studies in Perception and Action, which is essentially an abstract book. See also Russell & Sternad (2001) for intermittent control or synchronization in slower oscillations and Wolpert, Miall et al (1992) Evidence for an error deadzone (1992) or Park, Marino, Charles et al (2017) Moving slowly...
As already addressed in the answer to the detailed comment **#1** of this letter, we have carefully revised our references and exchanged some with the sources mentioned by the Reviewer.

49. **Lines 745-758:** Description of BS3 Control Strategy. It appears that BS3 is an inspired or "softened" form of a true bang-bang controller in order to make it more feasible for humans to employ. More explanation of BS3 needs to be included to explain how this version is different from a true bang-bang controller with discrete on-off transitions.
The baseline strategy *BL3* (formerly *BS3*) used for comparison of the experimental data modeled a bang-bang with a deadzone, which is a common implementation of this controller with three states instead of two [63]. To avoid confusion, this fact is now specifically mentioned in the manuscript (Line 753). Despite the implementation with a deadzone, it needs to be mentioned that the deadzone is very small in the investigated setup. It is only triggered in the short time frame when the pendulum link passes the motor link, resulting in a torque value τ that is low enough to lie below the threshold ϵ_T. Thus, although the modeled controller incorporates a deadzone, the behavior is mostly similar to a two-state bang-bang with only on-off transitions. We hope this clarifies the Reviewer's concern.

50. **Line 770:** replaced 'attached to' with 'connected to'
We have rephrased the sentence as suggested (Line 772).

51. **Line 776 onwards:** clumsy way of expressing the simple fact that the distance between the turning point location and the target determined what counted as a hit (+1) or overswing (-1).
We have rephrased the sentence as suggested (Ln. 776-780) and also specifically point out that neither *overshoots* nor *undershoots* changed the score, as both resulted in adding 0 points.

52. **Line 789-791:** 'For this comparison, only the periods where the target was hit on both sides were considered since only then was the correct energy level maintained.' What is the proportion of periods where the target was hit on both sides?
As suggested, we have added the number of *overshoots* and *undershoots* for each pendulum configuration and experiment variation in the Tables 1-3.

53. **Line 798:** gait and motion capture analysis is not the same category: you use motion capture to perform gait analysis .. this reads funny
We have rephrased the sentence to be less confusing (Line 807).

54. **Line 820:** as phase lag becomes a prominent metric, some more technical information for its calculation is warranted
We now introduce the phase lag as metric clearly in the Introduction (Ln. 99-102) and give more detail about the computation in Lines 825-834.

Statistics

1. How many data points per experimental configuration are entered into your analyses? The degrees of freedom in the t-tests in the results indicate that the analyses on 20 points (=20 participants). It may be useful to keep the individual trials or even the individual cycles in the analyses to represent the variance across trials.
The Reviewer's interpretation of the data points is correct. We have verified that the mean and standard deviation within-subject are very low, indicating that the chosen control strategy per participant remained similar and of low variance throughout the trials. This justified the validity of merging the three best trials of each participant into one large trial in the analysis. For this merged trial, we split the data into sections containing exactly one period of the pendulum motion and overlayed those sections of the same length to extract a mean curve per participant. The resulting sample sizes ($n = 20$ for Exp. 1 and 3, $n = 10$ for Exp. 2) are now specifically stated for each pendulum configuration and experiment variation in Tables 1-3. This evaluation motivated us to focus on investigating whether different control strategies between subjects emerged instead of analyzing the within-subject variation.

2. A t-test was used to test each hypothesis. In this case, the p-values need correcting depending on the number of hypotheses tested (Tukey or Bonferroni correction). The more hypotheses are tested, the higher the type I error, and the p-value needs to be lowered.
As already addressed in the answer to the detailed comment **#7**, the p-value was not corrected according to Bonferroni as it would have uncommonly worked in favor of our hypothesis. However, we acknowledge the valid criticism the Reviewer mentions and now specifically address the p-value adjustment and its implications in the method section (Ln. 857-863).

3. Clarify in the methods what software and what libraries/toolboxes/packages were used for data analysis and for statistical testing.
All data was analyzed with MATLAB2020b including the statistical analysis, which is mentioned in the relevant manuscript sections (Line 765, 784 and 841). Additionally, we now mention the specific functions that we have used for the *DTW* computation for the mode metric η and the phase lag calculation. Furthermore, all participant data and the complete analysis code will be publically available in *figshare*, such that readers can recreate the manuscript plots and inspect the used functions and methods.

4. **Line 837:** the same distance from the ideal NNM..." same compared to what?
The sentence has been rephrased to clarify that the distance of the individual baseline strategies *BL1-3* from the NNM was expected to be similar to the η-value obtained for the participants if one of the strategies underlie the human control approach (Line 852).

5. **Line 842:** what is the 'dedicated way in which the joystick handle was moved' what does dedicated mean?
We apologize for the confusing phrasing. "dedicated" should only emphasize the individually applied strategy per participant. We have reformulated the sentence to be less vague (Line 864).

6. **Lines 839-841:** "Again, the two-sided one-sample t-test was applied to test the null hypothesis that each baseline strategy leads to a similar pendulum motion path close to the ideal NNM as the strategy applied by the participants." Report the correlation method Pearson correlation, or was it regression? In lines 179-192, ensure that all four correlations have r-values and p-values reported. Clarify whether you report a slope + p-value (Linear Regression / Linear Model) or a correlation + p-value, as there are different statistical tools for determining the significance of slope and correlation coefficient.
We thank the Reviewer for pointing out how to improve our result reporting. We now specifically mention that the MATLAB function `corrcoef` was used, which caluclates the Pearson correlation (Line 867). Additionally, we ensured that all four correlations are reported with their r-values in the Results section (Ln. 217-223).

References

[2] Raftery A, Cusumano J, Sternad D. Chaotic frequency scaling in a coupled oscillator model for free rhythmic actions. Neural computation. 2008;20(1):205–226.

[4] Kugler PN, Turvey MT. Information, natural law, and the self-assembly of rhythmic movement. Routledge; 1987.

[8] Maurice P, Hogan N, Sternad D. Predictability, force, and (anti) resonance in complex object control. Journal of Neurophysiology. 2018;120(2):765–780.

[13] Latash ML. Anticipatory Control of Voluntary Action. Motor control. 2010; p. 1.

[14] Razavian RS, Sadeghi M, Bazzi S, Nayeem R, Sternad D. Body Mechanics, Optimality, and Sensory Feedback in the Human Control of Complex Objects. Neural computation. 2023;35(5):853–895.

[16] Nasseroleslami B, Hasson CJ, Sternad D. Rhythmic manipulation of objects with complex dynamics: predictability over chaos. PLoS computational biology. 2014;10(10):e1003900.

[22] Aiple M, Smisek J, Schiele A. Increasing impact by mechanical resonance for teleoperated hammering. IEEE transactions on haptics. 2018;12(2):154–165.

[31] Russell D, Hogan N. Dealing with constraints: A biomechanical approach. In: Images of the Twenty-First Century. Proceedings of the Annual International Engineering in Medicine and Biology Society. IEEE; 1989. p.892–893.

[32] Ohta K, Svinin MM, Luo Z, Hosoe S, Laboissiere R. Optimal trajectory formation of constrained human arm reaching movements. Biological cybernetics. 2004;91:23–36.

[33] Ben-Itzhak S, Karniel A. Minimum acceleration criterion with constraints implies bang-bang control as an underlying principle for optimal trajectories of arm reaching movements. Neural Computation. 2008;20(3):779–812.

[46] Hatsopoulos NG, Warren Jr WH. Resonance tuning in rhythmic arm movements. Journal of motor behavior. 1996;28(1):3–14.

[63] Song G, Agrawal BN. Vibration suppression of flexible spacecraft during attitude control. Acta Astronautica. 2001;49(2):73–83.

[2] Dotov DG, Stephen DG, Frank TD. Variability of Uni-Manual Pendulum Oscillation at and away from Resonance. In: Studies in Perception and Action X: Fifteenth International Conference on Perception and Action. Psychology Press; 2009. p. 8.

[5] Riley MA, Turvey MT. Variability and determinism in motor behavior. Journal of motor behavior. 2002;34(2):99–125.

[13] Kuznetsov AP, Migunova NA, Sataev IR, Sedova YV, Turukina LV. From chaos to quasi-periodicity. Regular and Chaotic Dynamics. 2015;20:189–204.

[26] Lakatos D, Petit F, Albu-Schäffer A. Nonlinear oscillations for cyclic movements in variable impedance actuated robotic arms. In: Proceedings - IEEE International Conference on Robotics and Automation; 2013. p. 508–515.

[28] Boulgouris NV, Plataniotis KN, Hatzinakos D. Gait recognition using dynamic time warping. In: IEEE 6th Workshop on Multimedia Signal Processing, 2004. IEEE; 2004. p. 263–266.

[41] Nagengast AJ, Braun DA, Wolpert DM. Optimal Control Predicts Human Performance on Objects with Internal Degrees of Freedom. PLOS Computational Biology. 2009;5(6):1–15.

[42] Seidler RD, Noll DC, Thiers G. Feedforward and feedback processes in motor control. Neuroimage. 2004;22(4):1775–1783.

[43] Hogan N. The mechanics of multi-joint posture and movement control. Biological cybernetics. 1985;52(5):315–331.

[45] Bloch AM, Leonard NE, Marsden JE. Controlled Lagrangians and the stabilization of mechanical systems. I. The first matching theorem. IEEE Transactions on automatic control. 2000;45(12):2253–2270.

[61] Paliwal KK, Agarwal A, Sinha SS. A modification over Sakoe and Chiba's dynamic time warping algorithm for isolated word recognition. Signal Processing. 1982;4(4):329–333.
