# Supplementary material for: Finding the rhythm: Humans exploit nonlinear intrinsic dynamics of compliant systems in periodic interaction tasks
Source: PLoS Comput Biol. 2024 Sep 3;20(9):e1011478. doi: 10.1371/journal.pcbi.1011478 (PMC11398697; doi:10.1371/journal.pcbi.1011478)
Supplement: Attachment — Submitted filename: ReviewAnswer.pdf [file pcbi.1011478.s005.pdf]

Annika Schmidt (an.schmidt@tum.de)
Technical University of Munich - CIT (I23)
Friedrich-Ludwig-Bauer Str. 3
85748 Garching

15th July 2024

PLOS Computational Biology
Academic Editor Prof. Dr. Bard Ermentrout
Section Editor Prof. Dr. Daniele Marinazzo

Response to the Reviewers for the Article Submission
"Finding the rhythm: Humans exploit nonlinear intrinsic dynamics of compliant systems in periodic interaction tasks"

Dear Editors, dear Reviewer,

we are very grateful that you repeatedly took the time to review our manuscript and provided us with very high-quality constructive feedback. This allowed us to increase the quality of our manuscript significantly from the first version to the current one. We appreciate your recognition of our previous efforts to address all your comments. After addressing your remaining concerns, we hope that you will find our current manuscript acceptable for publication in *PLOS Computational Biology*. We believe it will be a valuable contribution to the readership of this journal.

In the following, we will first address the Editors' comments and then the Reviewer's comments point-by-point. Line references generally refer to the clean version of the manuscript, but since significantly fewer text changes were required in this round of Review, we will additionally point out the lines for the version with tracked changes in red. As before, in the manuscript version with tracked changes, deletions are pointed out in red, and text additions are marked in blue.

Editors - Prof. Dr. Bard Ermentrout and Prof. Dr. Daniele Marinazzo

Thank you very much for submitting your manuscript "Finding the rhythm: Humans exploit nonlinear intrinsic dynamics of compliant systems in periodic interaction tasks" for consideration at PLOS Computational Biology. As with all papers reviewed by the journal, your manuscript was reviewed by members of the editorial board and by several independent reviewers. The reviewers appreciated the attention to an important topic. Based on the reviews, we are likely to accept this manuscript for publication, providing that you modify the manuscript according to the review recommendations.

I am going to ask for another revision, but if the reply to the reviewers is sufficiently detailed, I will not send it out for further review. What I would like to see is (1) all the typos should be fixed. (2) I find the paper readable, so how much you rewrite the text is up to you.

We thank you for the extended time and attention that our manuscript received, which helped us to successively improve it. After including the remaining recommendations of Reviewer #2, we believe the manuscript addresses all your required points, and we would be happy if it can now be accepted for publication in *PLOS Computational Biology.*

Additionally, I will quote here another editor regarding the statistics: "There's no reason to do an ANOVA with posthoc Tukey if your hypotheses involve individual t tests, you can go on with these, reduce the fishing expedition vibe, and increase the power. Also, correction for multiple comparison is also necessary for multilevel exploratory ANOVA (https://link.springer.com/article/10.3758/s13423-015-0913-5). The fact that the authors say: "we don't want to apply Bonferroni since this would even favor our own hypothesis" is not accurate, the correction to the alpha level is for evidence against the data being distributed according to the null hypothesis, regardless whose hypothesis this is. Now, frequencies are not independent, the spectrum is somewhat a continuum, so Bonferroni is probably too strict, False Discovery Rate or Benjamini Hochberg would be more appropriate."

We agree with the Editor's point that an ANOVA and post-hoc Tukey testing might not have been needed for our experiment. Nevertheless, we have decided to still carry out the statistical analysis as suggested by the Reviewer, although we do not report the ANOVA findings as part of the results. Instead, we mention it in the method section (Ln. 864-868, Ln. 921-927) and added *Supplementary information* with tables that summarize all statistical tests. We have also streamlined the methodology of our statistical analysis using the t-test, as we now consequently compared all metrics for all experiments (Exp. 1-3) statistically. For this, we have consequently applied the Bonferroni correction and refrained from mentioning our reasons for not using it. Although only relevant statistics are mentioned in the results section, again tables in the *Supplementary information* are provided for the carried out statistics, including all metrics for all baseline strategies in all experiments. We believe that with this addition, our statistics are methodologically sound and scientifically justifiable.

In addition to revising and extending the statistics methods and reporting, the new manuscript includes the following major changes:

- The abstract has been slightly modified, specifically the last paragraph.
- The intial presentation of the experiment procedure in the results section has been improved.
- Figure 4 has been revised as suggested by the Reviewer.
- "upper" and "lower" pendulum link has been changed to "first" and "second" link.
- The motivation for testing Experiment 3 together with Experiment 1 has been improved.
- Dr. Bernhard Weber has been added to the author list as he supported the final statistical analysis.

Reviewer #2

I applaud the authors for their efforts to address our admittedly very extensive comments. The manuscript is vastly improved compared to the last 2 versions. And yet, I wanted to provide suggestions on several more points detailed below. Especially the statistical analyses are still in need of improvement. Below are my remaining suggestions.

We once more want to thank the Reviewer for taking the time to provide us with such detailed and constructive feedback. We agree that addressing the long list of previously raised concerns and comments has significantly improved the quality of our manuscript, and we are happy that the Reviewer appreciates our efforts. We are convinced that addressing the remaining comments of the Reviewer will once more improve the quality of our work and believe the manuscript then to be a valuable contribution to the community.

Major Comments

1. I still have an issue with the statistics as there is a multitude of t-tests, with or without corrections. Reporting of a multitude of t-tests is regarded as scientifically clumsy and prone to false conclusions. **1)** I understand that Reviewer 1 suggested a distinction between a null hypothesis and alternative hypotheses. However, after much discussion with my group and colleagues, I strongly believe that this distinction is not suitable here. The authors want to test three baseline strategies and the NNM for how they account for the data. The expectation is that BL1 will 'win' in that there will be no significant differences between the human data and the BL1. Despite this expectation, the aim of testing a hypothesis is to falsify it. If it cannot be falsified, while others can be falsified, here BL1, BL2 and BL3, then this is regarded support for the NNM hypothesis. I think that each of the three BL1, BL2, and BL3 hypotheses should be put to test equally.
   2) Bonferroni corrections, if applied to few targeted pairwise comparisons is acceptable. However, your argument that these corrections should not be applied to all tests is not correct to my knowledge based on my first point.
   3) A simple solution to this testing issue is to do the following: for each of the four metrics conduct a one-way ANOVA for the subject values minus the values of BL1, BL2, and BL3. If there is a significant main effect, this is a first result stating that the three strategies are different from each other. Then, conduct posthoc pairwise comparisons (using Tukey corrections applied automatically in the statistics packages) to identify whether all pairs are different from each other. Then, perform a separate t-test to evaluate whether the subject data minus BL1 is different from zero.
   We understand the Reviewer's concerns and have addressed them in the new version of the manuscript taking into account each of the points raised by the Reviewer.

   1) We have revised our phrasing in the Introduction and refrain from explicitly calling *BL1* the *null hypothesis H0*. We still suggest that it is tested because it aligns with our hypothesis that humans intuitively excite the resonance frequency (Line 70, Line 71), while *BL2-3* resemble other control strategies known from human experiments (Line 73, Line 75). As suggested by the Reviewer, all baseline strategies are equally put to the test with the aim of falsifying them. The fact that *BL1* cannot be falsified in all comparisons is thus in line with our expectations and even extends them to suggest that a sine wave might not be a bad approximation for the underlying control approach of the human in the tested experiment.

   2) We agree with the Reviewer's argument and have adjusted the manuscript, such that the Bonferroni corrections are applied throughout for the pairwise comparisons. We refrain from the suggestion that it might now apply in the considered case.

   3) Although we believe that a one-way ANOVA might not have been needed in our specific experiment setup due to clear expectations, we agree with the Reviewer that their suggested approach is

methodologically better. Thus, we carried out a one-way ANOVA for the data of Experiment 1, followed by a post-hoc pairwise comparison using Tukey correction. We have adjusted the method section accordingly. We refrain from reporting the ANOVA findings in the results section as all comparisons were highly significant, but have added *Supplementary information* that state all statistics for the ANOVA and post-hoc comparisons. This fact is specifically mentioned in the method section (Ln. 864-868, Ln. 921-927). Additionally, we have extended the t-testing (now applying the Bonferroni correction) to consequently apply it to all relevant metrics: hit score, oscillation frequency, mode metric, phase lag, and deflection ratio. Although we only mention relevant values in the results section, we have added separate tables presenting all the statistics carried out for Experiment 1, 2 and 3 in the *Supplementary information*. The respective tables are pointed out in the results section (Lines 159, 183, 211, 246, 258, 289 , Lines 170, 196, 225, 270, 284, 220). We thank the Reviewer for this suggestion and believe our statistical comparisons have now become methodologically consistent and scientifically sound.

2. But please consider a perennial conundrum: why are NNM and BL1 considered separately, since BL1 is derived from the NNM?
We apologize for the unclear presentation of our results. The NNM is not compared as a separate strategy to the participant data but is only used as an ideal reference, especially for the pendulum trajectories that are excited by the participants and *BL1-3* quantified by the mode metric. Thus, in the cases where we applied or suggested system changes, we had to recompute the NNM for the changed system to be able to predict the changed pendulum trajectories and calculate the respective mode metrics. However, the NNM is *not* a comparative control strategy and is not treated as such. Nevertheless, we understand the origin of the possible confusion, especially when comparing the eigenfrequencies, due to the fact that the frequency applied by *BL1* was indeed derived from the predicted frequencies of the NNM. Thus, when comparing the oscillation frequencies excited by the participants, we were, of course, interested in the similarity to the ideally predicted ones of the NNM, but these are, in fact, identical to the ones of *BL1*. We now specifically point this out when comparing the frequencies to avoid confusion (Line 137, 154, Line 147, 164). We do compare the participant data to the predicted eigenfrequencies only for the comparisons with changed system dynamics and recomputed NNM. In these cases, no baseline strategies were applied, and the focus was solely on validating whether the intrinsic resonance was matched at all instead of analyzing how this was achieved.

3. The design of the experiments remains unclear: In line 125, the sentence says that each trial is 20s long, later it says 40s. It also states that all conditions are randomized. This cannot be correct. Please state what was done in Experiment 1, e.g., 4 trials each for P0 and P90, randomly shuffled. Then in Experiment 2: ..„ Experiment 3 … Also, were participants informed of pendulum change between experiments? What were they told?
We thank the Reviewer for pointing out the typo in line 125, which should indeed be 40 s. Throughout all experiments, the trials consistently lasted 40 s. We apologize for the confusion. As suggested, we have rephrased the pointed out paragraph starting in Line 115 (Ln. 228-243) to outline each experiment condition sequentially and also emphasize the given trial time and task instruction for each. We now also specifically state that participants were informed about the changes applied for Experiment 2. However, the task remained identical, and the expected implications it could have on the control strategy were not mentioned to the participants to avoid altering their intuitive control strategy. We believe the rewritten paragraph is much easier to follow now, thanks to the Reviewer's suggestions.

4. The correlations shown in Figure 4 are atypical as they include participant as a factor that is not needed for the questions asked: Is there a correlation between phase lag and number of hits, and

phase lag and the mode metric? The straightforward way to show the data is to directly plot phase lag against hits and phase lag against the mode metric. In each of the two plots you can include the data for P0 and P90 in different colors.
We thank the Reviewer for their suggestion and have revised Figure 4 accordingly. As suggested, we now plot the phase lag against the hit score (Fig. 4A) and the mode metric (Fig. B), combining *P0* and *P90* in one plot. Additionally, we have added a figure (Fig. 4C) that sorts the individually applied phase lags per participant for the *P0* and *P90* configuration to emphasize that participants were consistent in their applied control strategies as they applied a similar phase lag for both pendulum configurations.

5. Experiment 3 receives very little coverage. This is odd as it is a very important experiment that shows that also targets not matching the NNM can be hit. This important demonstration should be signaled better. The plausible conclusion is that wrist stiffness is adjusted to change the system behavior to match the new targets. It is only non-optimal that this human-added stiffness is tweaked into the simulations by changing k1. This does not correspond to the human-added stiffness. It is too much to extend the model, but this 'quick fix' should be commented on. In principle, it appears better to me to have used the same pendulum configurations and only changed one thing, the target locations
Mention that approximating the human stiffness with a change of k1 is not entirely correct, but a model extension would extend the scope of the paper.
We thank the Reviewer for the feedback on this point, and we take the opportunity to clarify it here and in the manuscript.
First, we would like to emphasize that the stiffness k_1 was not changed in any of the experiments. The spring stiffness was constant ($k_1 = 5\ \mathrm{N\,m\,rad^{-1}}$) in every interaction that the participants had with any of the double pendulum configurations.
Only *after* the experiments, we recomputed the NNM for pendulum systems with changed values of k_1 as a tool to help interpret the results from the user study. Although the Reviewer points out correctly that changing the value of k_1 is not specifically accounting for the human-added stiffness, we believe it was an acceptable 'quick fix'. The human arm stiffness, denoted here with k_h, and the spring stiffness k_1 were assumed to act in series, thus resulting in a combined stiffness k_{comb} of

$$k_{comb} = \frac{k_1 k_h}{k_1 + k_h} \,. \tag{1}$$

Therefore, it can be assumed that if the human changes the arm stiffness, the overall stiffness k_{comp} would change accordingly. Although we agree that changing the value of k_1 is only a very rough approximation for this behavior, it appeared to be a sensible approach to better interpret the data of the users, specifically for Experiment 2 with the changed target size and Experiment 3. Nevertheless, we agree with the Reviewer that this is only a very rough approximation and mention this specifically in the manuscript now in (Ln. 434-440, Ln. 469-475). Additionally, we revised the abstract to stress the importance and findings of Experiment 3.

6. The labeling is sometimes odd. For example, reference to the upper link and upper spring of the pendulum is misleading as the pendulum is not suspended in gravity and the first link points to the left.
We agree with the Reviewer that the terms "upper" and "lower" might be misleading in our experiment arrangement as the pendulum is indeed not suspended in gravity. We took up the Reviewer's suggestion and used another naming convention. Instead of "upper", we now refer to the link attached to the joint on the wall as "first link", making the "lower link" consequently the "second link". To make the naming even clearer, we added the parameter values l_1 and l_2 to Figure 1 and mentioned those parameter names when first introducing the links..

7. The experimental set-up is clear now. But for future studies, I wanted to make one comment: in the virtual implementation, the handle overlies the double pendulum, which in this constellation is physically impossible to manipulate. The hand and the upper first link of the pendulum coincide. I would regard much more intuitive of the handle was non-overlapping and pointing to the right of the system.
We agree with the Reviewers observation that overlaying the input handle with the upper pendulum link cannot be realized in a physical setting and might thus be unintuitive. We would still like to outline our inital reasoning for this choice as we had also considered the option you suggest: When designing the study, we believed it would make it easier for users to understand how their handle motion affected the upper pendulum link. In the chosen configuration, moving the handle in one direction pulled the pendulum link in the same direction through the spring coupling as can be seen in the figure below on the left. When arranging the handle to the opposite side, it still needs to be coupled to the upper pendulum link, e.g., as seen in the below figure on the right. In this case, moving the handle in one direction would result in a mirroring effect for the pendulum motion. For this reason the presented handle arrangement was chosen as it appeared more intuitive to us at the time. However, having gained experience with users in the experiment setup now, we are convinced that humans would just as quickliy understand the pendulum motions in the arrangement you suggest and we will follow your recommendation in future experiments.

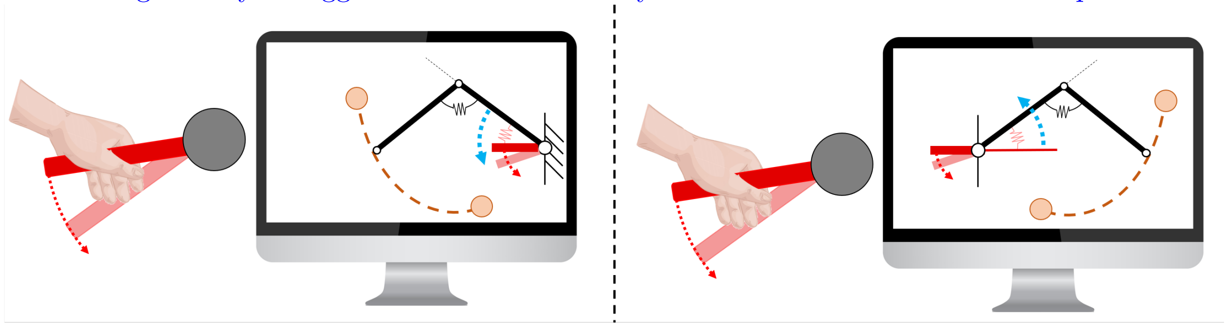

The descriptions are still very wordy. In addition, there is a lot of duplication between the results and the methods. In this order of results and methods (which I think is not helpful), the result section needs some wording to make the results understandable, including the motivation of experimental and analysis choices. The methods should simply describe the methods without any further embedding into the scientific rationale. In the current version, the methods contain all the motivation and reasoning, which makes them overly long and repetitive. For example, the BL3 strategy should simply be described: a bang-bang controller was used of the following form .. eqs 8 and 9.
We agree with the Reviewer's opinion that some content duplications appear between the results and the methods. We believe this largely stems from our decision at the inital manuscript preparation to follow recommended organizational structure suggested on the website of *PLOS Computational Biology*. Due to our lack of prior experience with this journal, we followed the outline and presented the *Results* first. In hindsight, we agree that we should have presented the methods first as in our specific case the theoretical background is essential to understand the results sufficiently. While we will more carefully assess our chosen structure in the future to improve the reading experience for the reader, we hope the Reviewer agrees that the current version of our manuscript is very well readable and tolerate the remaining content duplications.

Minor Comments

1. Abstract: Last sentence not needed. This manuscript is not introducing NNMs. Therefore, this sentence is a misrepresentation of the current contributions and must be removed. Instead, I would mention that you conducted 3 experiment and Experiment 3 demonstrated that subjects could also hit target off the NNM, probably by stiffening their wrist.
   We have revised the second half of the abstract as suggested by the Reviewer. We now mention the three different experiment parts and emphasize on the human capability to hit the targets when they are not aligned with the NNM. Instead, we refrain from mentioning the introduction to NNM at the end.

2. Author Summary: "To verify if the excited motions match the actual intrinsic dynamics of the objects, nonlinear dynamics need to be computable for comparison." The English is not correct here.
   We thank the Reviewer for pointing out this sentence. We have rephrased it.

3. Line 12: "..bouncing a ball by supporting intrinsic system dynamics" Supporting is the wrong word here.
   As suggested by the Reviewer, we have replaced "supporting" with "tuning to" to emphasize the human tunes (their control) to the system dynamics.

4. Line 42: "This uniquely enables us to.." The word uniquely sounds odd.
   We have rephrased the sentence without using the word "uniquely" (Line 43).

5. Lines 189-191: Is the conversion in degrees needed?
   The conversion from radians to degrees is not needed for technical reasons but we believe it is more intuitive for most people. Most people have an intuition of how much 5° is, but cannot visualize 0.088 rad. Thus, directly stating the amplitudes in degrees aids readability.

6. Line 204: "..which is compared to the baseline strategies again closest to the values obtained for BL1." The word again here is wrong. The whole sentence structure is wrong but I am not sure exactly what the authors want to convey. The values for the mode metric has no defined scale, hence the absolute values are not meaningful. It may therefore be good to compare the values against each other with statistical comparisons.
   We agree with the Reviewer's comment that the sentence structure was odd and the meaning therefore confusing. We have rephrased the sentence, which should clarify the meaning (Ln. 207-210, (Ln. 220-225). Additionally, as mentioned in the answer to Major Comment #1, we have extended the statistical comparison now including all metrics. In the mentioned section, we now state the values for the statistical comparisons and also refer to the table in the *Supplementary information* summarizing all statistics (Tab. S3.

7. Line 440: The reference to the information bandwidth as in Fitt's Law is unclear and not needed.
   As suggested, we have removed the referral to the information bandwidth, which makes the sentence more concise. We thank the Reviewer for their suggestion

8. Line 589. Indicate the recording frequency
   The recording frequency was identical to the control update frequency of 1 kHz, which we now specifically point out (Line 598, Line 634).

9. Line 601. A parenthesis is lacking '(', and there is no unit of measure after the range of A.
   We have corrected the missing bracket spotted by the Reviewer. Since the unit of A is rad it was not specified as it is uncommon practise to note it. Nevertheless, to avoid confusion, we have now added rad as unit in Line 610 (Line 647).

10. Line 622: what is a high-score game?
    The term "high-score game" appeared intuitive to us, but we understand the Reviewer's point that this might not be a commonly used term and refrain from using it in Line 631 (Line 669). Instead, we stress that the goal is to achieve a high score, where score is the number of hits.

11. Line 652: group, r remained ... delete 'the'
12. Line 684: 'following' .. delete
13. Line 723: tested the investigated systems... delete 'on'
14. Line 787: delete 'attempts' in error attempts
    All the above sentences were corrected as suggested.

15. Line 694-701: very confusing description. It remains unclear whether each trial was from a different condition, or blocked. Exp 3 has the different targets? Why coupled with Exp 1? There must have been considerable breaks to change the parameters for Exp2?
    We understand that not only the description, but also the fact that Experiment 1 and 3 were tested in the same trial run appears confusing. The reason for this was that we did not want to bias the participants in their expectations and control strategies, such that we left them uninformed whether the targets were aligned with the NNM (*P0* and *P90*) or not (*P45*). By presenting all pendulum configurations in the same trial, we could randomize the appearance of the configurations per participant, while the task remained identical throughout the whole trial. This aided to limit learning effects and avoid biasing the participants to suspect different conditions that might have affected them to change their control strategy. In contrast, the changes applied in Experiment 2 were very noticable for the participants. The increased system mass obviously influenced the system dyanamics and perceived haptic feedback, while the decreased target size is visually apparent. Thus, the participants were informed about the respective noticable changes. Additionally, it was necessary to recompile the system, which took a few seconds. This time was also used to give the participants some rest. Conclusively, two experiment runs where carried out, where the first tested the conditions of Experiment 1 and 3, while the second one tested the conditions of Experiment 2. The order in which the two trials were performed were randomized per participant, such that half the participants started with the augmented setup of Experiment 2, and the other half started with the run that tested the conditions of Experiment 1 and 3.
    We rephrased the wording in the results (Ln.127-132, Ln. 137-142) and the methods section (Ln. 681-684, Ln. 723-726) to clarify the arrangement and the reasoning for it.

16. Line 728-743: lengthy complex justification for BL2 which is a simple controller at half of the eigenfrequency: reliant on feedback and smooth, low effort, I find the three considerations confusing and conflicting, e.g. slower frequency is not necessarily low effort, and relying on feedback is not necessarily smooth.
    We agree that the introduction for *BL2* might be a bit lengthy. However, we wanted to provide literature-based background on why we chose to test a control strategy, where the excitation frequency was considerably lower than the eigenfrequency. In fact, the choice of half the resonance frequency was arbitrary and we could have chosen an even lower value. Nevertheless, we accept the Reviewer's comment that a low frequency does not always translate to low effort and smoothness. Thus, we rephrased the sentences to point out that in the specific case of our experiment, the lower excitation frequency entailed lower forces with smoother curves (Line 749, Line794).

17. Line 739. "The amplitude was once more tuned empirically to hit the targets." The authors should indicate the amplitude values.
    We thank the Reviewer for pointing this out. The amplitude was set to $A = 1.0$ rad, which is now in the text (Line 753, Line 799).

18. Line 776-780: this allocation of points including subtraction of points contradicts what was written higher up in the results.
    It appears that we have not stressed enough that neither an *undershoot* nor an *overshoot* changed the hit score during the trials. The hit score during the trials only increased when a target was correctly hit. The allocation of points for the two error conditions was exclusively used for post-processing as giving the two errors different values helped to align recording tracks and easier identify which error occurred. Only for this analysis, it became relevant that a value of -1 was allocated to an *overshoot* and 0 identified an *undershoot*. However, it appears that mentioning the respective points for the two error occurances is still confusing, such that we now refrain from mentioning them in the manuscript in Line 792 (Line 837).

19. Line 855: this is problematic: a statistical test aims to falsify, to not show a difference can be due to noise in the data. The argument for omitting Bonferroni corrections is therefore sketchy. See above for thoughts on a simple analysis strategy.
    As detailed in the answer to the Major Comment #1, we have revised our statistical analysis and adjusted the methods section accordingly. We refrain from our previous reasoning and consequently apply the Bonferroni correction to the statistical analysis putting all baseline strategies *BL1-3* equally to the test.
